# Remotely Delivered Nutrition Interventions with or Without Physical Activity Interventions in Adults with Cardiovascular Diseases: A Systematic Review of Randomized Controlled Trials

**DOI:** 10.3390/healthcare14162523

**Published:** 2026-08-13

**Authors:** Maria Dimopoulou, Maria Isakoglou, Jonathan Hoes, Odysseas Androutsos

**Affiliations:** 1 Laboratory of Clinical Nutrition and Dietetics, Department of Nutrition and Dietetics, University of Thessaly, 42132 Trikala, Greece; jonathan.hoes@telenet.be; 2 Clinical Exercise Physiology and Rehabilitation Laboratory, Physiotherapy Department, School of Health Sciences, University of Thessaly, 35132 Lamia, Greece; misakoglou@uth.gr

**Keywords:** advice, cardiovascular disease, nutrition, intervention, physical activity, remote rehabilitation, telehealth

## Abstract

**Highlights:**

**What are the main findings?**

- Remote interventions may improve disease management among patients with cardiovascular diseases.
- Remote interventions are associated with significant improvements in diet quality and favorable changes in physical activity levels.

**What are the implications of the main findings?**

- Remote care improves healthcare accessibility for patients with cardiovascular diseases, particularly those residing in geographically remote areas or experiencing social vulnerability, thereby supporting more equitable and personalized care delivery.
- Remote nutritional and physical activity interventions represent a promising and feasible approach to the long-term management of chronic cardiovascular diseases.

**Abstract:**

**Objective:** The long-term management of cardiovascular diseases (CVDs) requires comprehensive rehabilitation strategies aimed at optimizing functional recovery and promoting behavioral changes. These interventions may include digital technologies that emerged as promising approaches to facilitate lifestyle changes, such as dietary behavior and physical activity (PA). **Aim:** This review aimed to synthesize the current evidence for the potential impact of remotely delivered nutrition interventions with or without a combination of PA interventions on biochemical biomarkers, cardiovascular indexes, anthropometric parameters, levels of PA, exercise and functional capacity, adherence to the Mediterranean Diet (MD), diet quality and quality of life, rehospitalization, urgent visits, death and acute events in adults (≥18 years) with CVDs. Secondary outcomes were accessibility, safety, usability, and adherence. **Methods:** The PubMed, Web of Science, and Scopus databases were comprehensively searched up to 2026 for Randomized Controlled Trials (RCTs) published in English, following the Preferred Reporting Items for Systematic Reviews and Meta-Analysis (PRISMA) guidelines. The methodological quality was assessed using the revised Cochrane Risk of bias tool for RCTs (RoB2). **Results:** 11 RCTs met the inclusion criteria. Sample sizes ranged from 66 to 879 participants. The duration of the interventions varied from 4 weeks to 12 months. The interventions were delivered remotely using a range of digital health technologies, including web-based programs, mobile phones, wearable devices, smartphone applications, and mini-apps. Favorable changes were observed in diet quality and in adherence to MD and PA levels, with no intervention-related adverse events reported. The RoB2 assessment indicated that the majority of included studies (6 out of 11) were classified as having some concerns regarding risk of bias. **Conclusions:** Remotely combined nutrition and PA interventions are a feasible and effective approach for improving mainly patient-reported outcomes in adults with CVDs. These findings support integrating remote multimodal rehabilitation into routine cardiovascular care and highlight the potential of telehealth-based models to enhance patients’ access to rehabilitation services.

## 1. Introduction

Cardiovascular diseases (CVDs) remain among the leading causes of morbidity, disability, and mortality worldwide [1]. Conditions such as chronic heart failure (HF), and coronary artery diseases (CADs) are associated with substantial symptom burden, frequent hospitalizations, and reduced quality of life (QoL) [2]. In the aforementioned pathologies, lifestyle factors, particularly physical inactivity and suboptimal nutrition, play a critical role in disease progression, functional decline, and adverse clinical outcomes [3].

Nutrition encompasses the consumption of a balanced diet and supports a lifelong commitment to healthy eating habits [4]. Physical activity (PA) is defined as any movement of the body produced by skeletal muscles that results in energy expenditure [5]. Both PA and dietary modification are well-established components of comprehensive management for CVDs [6]. Participation in cardiac rehabilitation (CR) programs has been shown to improve exercise capacity, symptom control, cardiopulmonary function, and health-related QoL, while reducing hospital re-admissions [7]. Likewise, nutrition interventions, including heart-healthy dietary patterns, weight management strategies, and targeted nutritional supplementation, further improve metabolic and functional outcomes [8]. Despite the proven evidence supporting their efficacy, participation in traditional, conventional center-based rehabilitation and nutritional programs remains suboptimal. Common barriers include patients’ limited mobility, transportation challenges, comorbidities, work or caregiving responsibilities, and geographic distance from specialized centers [9].

The remote delivery of diet and exercise interventions has emerged as a promising strategy to overcome these barriers [10]. Advances in telemedicine, mobile health technologies, wearable sensors, and web-based platforms have enabled the delivery of structured lifestyle interventions directly to patients in their home environments [11]. These interventions may include supervised or unsupervised exercise training, virtual nutritional counseling, real-time physiological monitoring, and behavioral support through digital tools [12,13,14]. The rapid expansion of telehealth during the COVID-19 pandemic further accelerated the implementation of these care models, highlighting their feasibility and potential scalability in the long-term chronic disease management [15].

Growing evidence suggests that remote nutrition and PA advice interventions may yield clinical benefits comparable to those of traditional in-person programs in selected patient populations [16]. Additionally, remote models may have the potential to improve access and increase participation, particularly among older adults and individuals living in rural or underserved areas [15]. Although remote nutrition interventions have shown increasing promise in cardiovascular prevention and management [17], remote nutrition interventions with or without PA interventions in patients with CVDs have not been extensively studied. Moreover, the available literature characterized by heterogeneity in intervention design, digital technology tools, duration, and assessed outcome measures, limits the generalizability of findings. Furthermore, implementation challenges related to digital literacy, long-term adherence, safety monitoring, and integration into routine clinical care have not been fully addressed [18]. Given that the available evidence remains fragmented, no comprehensive synthesis has specifically examined their role in delivering nutritional care across the spectrum of CVD.

Therefore, the present review aims to synthesize the current evidence regarding the effectiveness of remotely delivered nutrition interventions with or without PA interventions on clinical outcomes patient-reported outcomes in adults with CVDs, while identifying existing knowledge gaps and priorities for future research.

## 2. Methods

### 2.1. Data Sources and Search Strategy

This systematic review was conducted in accordance with the methodological recommendations of the Cochrane Collaboration and reported according to the Preferred Reporting Items for Systematic Reviews and Meta-Analyses (PRISMA) statement [19]. A literature search was conducted via PubMed, Web of Science, and Scopus, using relevant keywords and the Boolean operators “OR” and “AND” from inception up to 12 January 2026. The search strategy was developed a priori before the review was conducted, independently reviewed, and verified by all authors. The selection of these databases was based on their reputability, comprehensiveness, and relevance to healthcare and medical research (Supplementary Table S1). The complete search strategies for all databases are provided in Supplementary Table S2. No protocol was registered or published before conducting this review. A complete checklist, according to the PRSMA statement, is reported in Supplementary Table S3. This systematic review was registered with PROSPERO (7 August 2026).

### 2.2. Eligibility Criteria

This systematic review included RCTs published in English and targeted at adults with CVDs (CADs, acute and chronic HF, CABS: coronary artery bypass surgery, CHD: coronary heart disease, PCI: percutaneous coronary intervention; ACS: acute coronary syndrome, and/or mixed populations) that participated in remote nutrition and/or PA interventions. Eligible studies evaluated remotely delivered nutrition and/or PA interventions. A remote nutrition intervention was defined as the remote delivery of diet and/or nutritional counseling, education, coaching, or behavior change support through telehealth platforms, mobile applications, wearable monitoring devices, telephone, or other digital communication technologies, with the aim of improving dietary behaviors and cardiovascular health outcomes. Interventions incorporating both nutrition and PA components were considered multimodal remote lifestyle interventions. These interventions were analyzed separately from nutrition-only interventions whenever the available data permitted. Participants in the intervention group (IG) (solely advice and/or constructed interventions) received dietary and nutritional counseling/advice/coaching, with or without a combination of PA. Participants in the control group (CG) received in-person diet, nutritional counseling, education, coaching, or behavior change, with or without a combination of PA, usual care (e.g., pharmacological therapy), education, or no intervention. For the purpose of this review, nutrition was defined as the process of utilizing food for growth, metabolism, and repairing tissues [4]. Diet was defined as the intake of food and drink, customized for each person from day to day and planned to meet specific requirements [4]. PA was defined as any movement of the body produced by skeletal muscles and results in energy expenditure while structured exercise is defined as a planned and supervised exercise regimen with prescribed exercise frequency, intensity, duration, and progression [5]. The primary outcomes of interest were biochemical biomarkers, total cholesterol, low-density lipoprotein-cholesterol (LDL-C), high-density lipoprotein-cholesterol (HDL-C), triglycerides, and non-high-density, lipoprotein-cholesterol, glucose, glycated hemoglobin (HbA1c), cardiovascular indexes (heart rate, systolic and diastolic blood pressure), anthropometric parameters including body mass index (BMI), waist circumference, waist-to-hip ratio, body composition, levels of PA, exercise and functional capacity, adherence to the Mediterranean Diet (MD), diet quality, QoL, rehospitalization, urgent visits, death and acute events. Furthermore, feasibility outcomes were assessed across several domains, including implementation, demand, acceptability, practicality, safety, and clinical utility [20]. Safety was defined as the occurrence of unintended physical injury resulting from or contributed to by study participation, while clinical utility referred to the practical usefulness of the intervention, specifically the extent to which its results inform beneficial actions (e.g., treatments) that improve patient health outcomes [20]. These measures were considered secondary outcomes. The inclusion of only RCTs was intended to minimize the risk of bias and confounding associated with non-randomized study designs, thereby enhancing the methodological rigor, reliability, and comparability of the synthesized evidence. Eligibility criteria were structured according to the PICOTS framework, considering population, intervention, comparator, outcomes, time frame, and study design characteristics. The predefined eligibility criteria are summarized in Supplementary Table S4. Studies involving animals, structured exercise programs, were not RCTs (systematic reviews, observational studies, case studies, conference abstracts, unpublished material), were published in languages other than English and before 2011, included mixed populations or patients with other diseases, or lacked full-text access were excluded from this review. The exclusion criteria were summarized in the Supplementary Table S5.

### 2.3. Study Selection, Data Extraction, Synthesis of Data

Firstly, all retrieved studies were imported into the Rayyan software package (https://rayyan.ai). After duplicate records were removed, two reviewers (M.D. and M.I.) independently screened titles and abstracts of the retrieved articles for eligibility criteria, after duplicates were removed, and the full texts of the potentially relevant ones. The full-text articles were assessed, and the reasons for exclusion were recorded. Any disagreements between the two reviewers were initially resolved through discussion and consensus. When consensus could not be reached, a third reviewer (O.A.) was consulted to reach a final decision. Inter-reviewer agreement was not formally assessed. Then, two reviewers (M.D. and M.I.) independently extracted data using a prespecified form with the following domains: authors, publication year, country, population disease, sample size (age), female participants, technology/digital tools, IG (participants’ number, type of intervention, program duration), CG (participants’ number, type of intervention, program duration), outcome measures, key findings. The findings were then compared across studies to identify recurring similarities and differences regarding the aforementioned outcomes of remote interventions in adults with cardiovascular disease. Discrepancies at any stage were resolved by a third reviewer (O.A.). In cases of data unavailability, attempts were made to contact the authors. The findings of the included RCTs were synthesized qualitatively due to heterogeneity in study populations, intervention characteristics, outcome measures, and reporting approaches. A meta-analysis was not performed because substantial methodological heterogeneity was observed across studies, including variations in intervention delivery methods, duration, comparator groups, and outcome assessment. Therefore, a qualitative synthesis was considered the most appropriate approach to summarize and interpret the available evidence.

### 2.4. Risk of Bias in Individual Studies

The risk of bias of the included RCTs was assessed independently by two reviewers (M.D. and M.I.) using the Cochrane Risk of Bias tool for RCTs (RoB2). The RoB 2 tool evaluates five domains of bias: the randomization, deviations from the intended interventions, missing outcome data, measurement of the outcome, and selection of the reported result. Based on these domains, each study was assigned an overall judgment of low risk of bias, some concerns, or high risk of bias [21]. Any disagreements between the reviewers were resolved through discussion and consensus. When consensus could not be reached, a third reviewer (O.A.) was consulted to reach a final decision.

## 3. Results

### 3.1. Search Results

**Figure 1:**
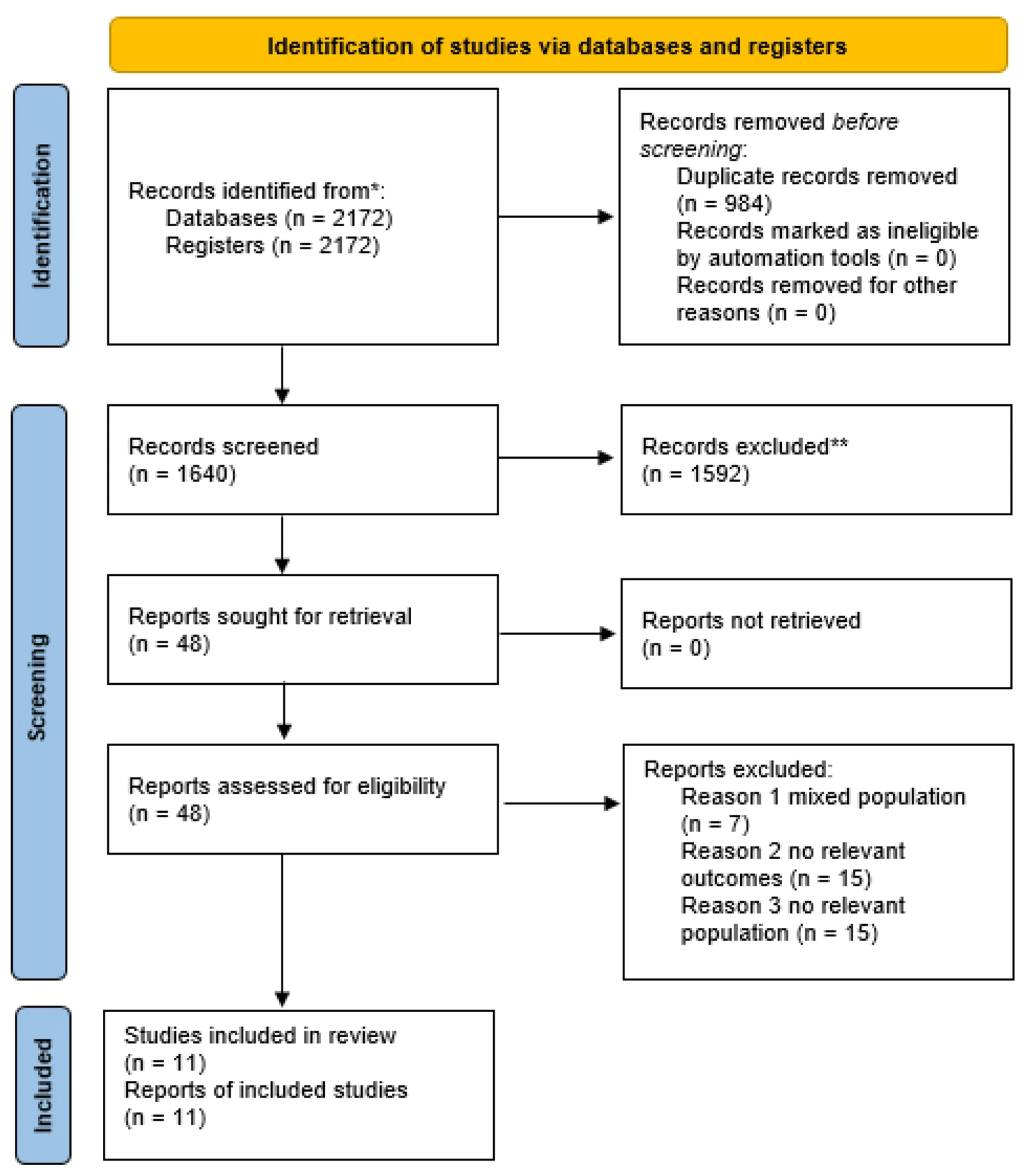
Prisma flow chart of the study (version 2000) [22,23,24,25,26,27,28,29,30,31,32]. * PubMed (*n* = 430), Web of Science (*n* = 1116), Scopus (*n* = 626), ** Not appropriate title/abstract.

A total of 2172 records were identified through three databases. After duplicates were removed and title/abstract screening, 11 RCTs [22,23,24,25,26,27,28,29,30,31,32] published between 2011 and 2026 met the inclusion criteria and were included in this review. The study identification, screening, eligibility assessment, and final inclusion process are illustrated in the flow diagram (Figure 1).

### 3.2. Characteristics of the Included Studies

Three [22,23,24] of the included studies focused exclusively on nutrition interventions, while the remaining studies combined nutritional components with PA interventions. In the CG, nine studies [22,23,25,26,28,29,30,32] used usual care, whereas one study incorporated self-monitoring [27] and e-education [31] components.

Sample sizes ranged from 66 [24] to 879 [29] participants. The duration of the interventions varied from 4 weeks [24,27] to 12 months [28,31], while follow-up periods extended from 3 months [25,26] up to 5 years in one study [28].

The included RCTs were conducted across several countries, including Spain (*n* = 4) [22,23,26,30], China (*n* = 2) [25,32], Canada (*n* = 1) [31], the Republic of Korea (*n* = 2) [27,29], the Netherlands (*n* = 1) [26], Norway (*n* = 1) [28], and New Zealand (*n* = 1) [24]. Six studies were multicenter trials [22,24,26,27,29,31]. The dropout rate ranged from 3% [26] to 27% [25] during the intervention period and from 1% [22] to 14% [32] at the follow-up measurements.

The study populations included patients with a range of cardiovascular conditions. Three studies enrolled participants with CVDs [24,28,32], three studies included patients with CAD [23,26,30], two studies focused on CHD [25,29], two studies on HF [26,31], one study on acute HF [27], one study on hypertension [26], and one study on atrial fibrillation (AF) [22]. Two studies initiated during the hospitalization period [25,30]. The main characteristics of the included studies are summarized in Table 1 and Table 2.

### 3.3. Risk of Bias of Included Studies

**Figure 2:**
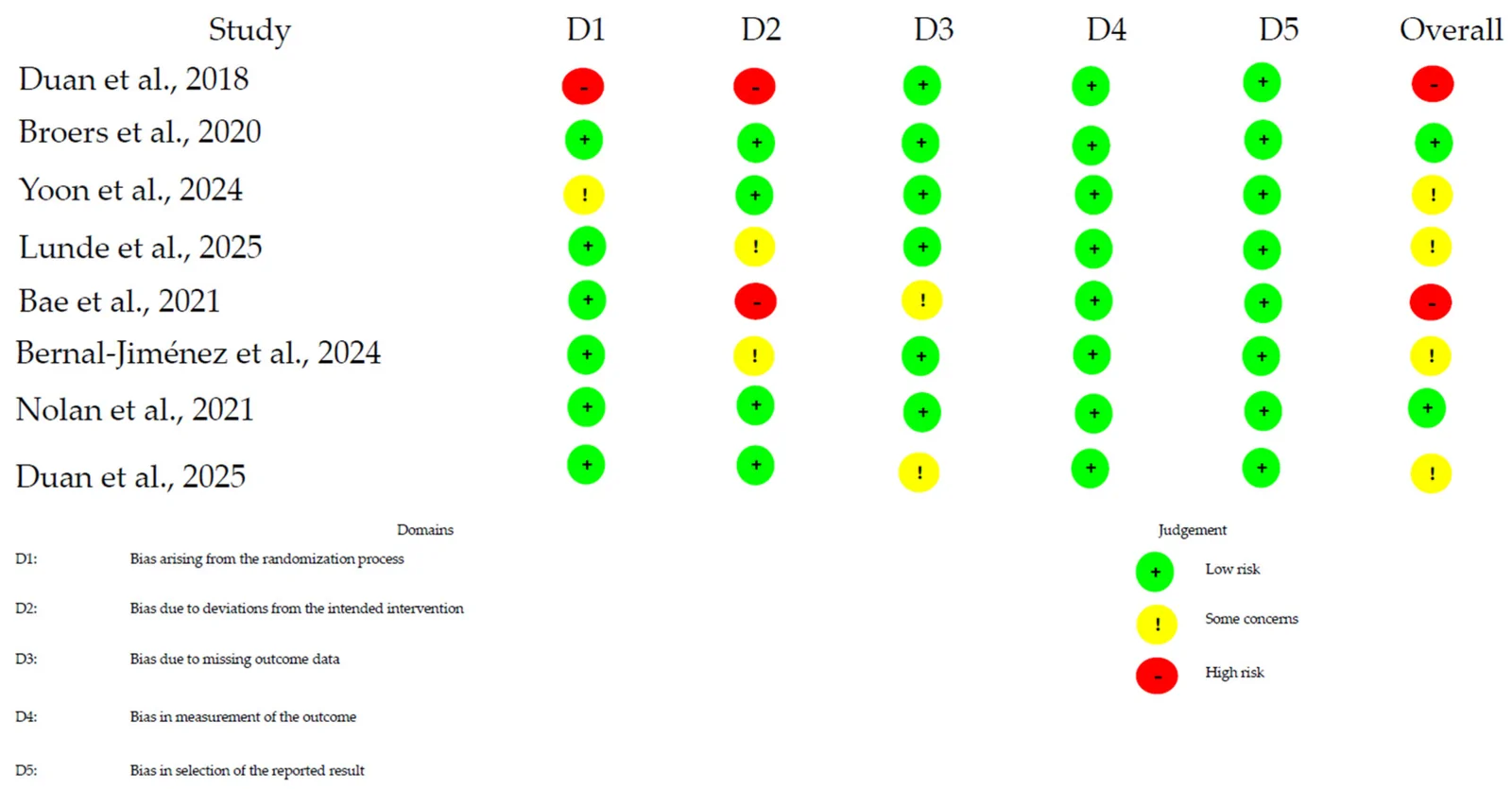
Risk of bias analysis. Summary of risk of bias assessment in the included studies combined remotely delivered nutrition and physical activity interventions [25,26,27,28,29,30,31,32].

**Figure 3:**
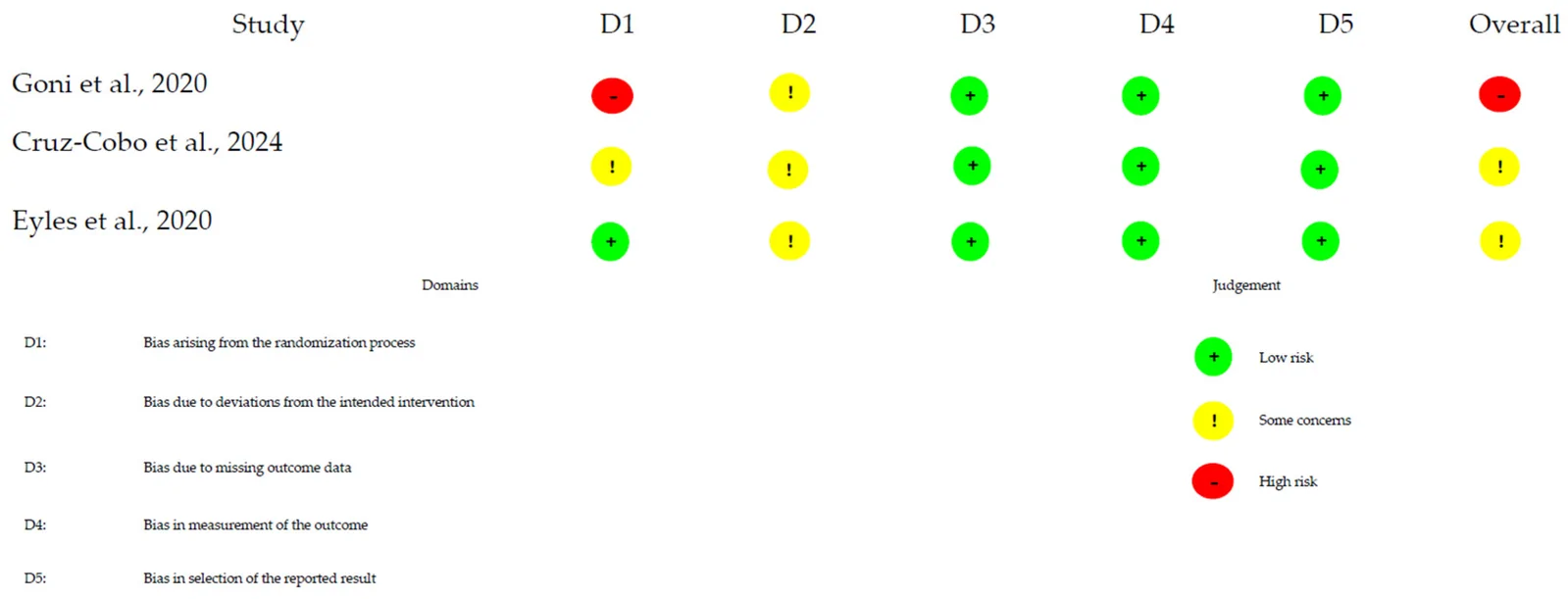
Risk of bias analysis. Summary of risk of bias assessment in the included studies remotely delivered nutrition interventions [22,23,24].

The risk of bias of all included RCTs was assessed using the RoB 2 tool. The overall risk-of-bias assessment demonstrated that two studies [26,31] were judged to be at low risk of bias, three [22,25,29] were classified as being at high risk of bias, and the remaining studies [23,24,27,28,30,32] were judged to raise some concerns. Referring to the risk of bias from the randomization process, two studies were judged to be low in seven studies [24,26,28,29,30,31,32], high in two studies [22,25], and associated with some concerns in two studies [23,27]. Bias due to deviations from the intended interventions was rated as low in four studies [26,27,31,32], as having some concerns in five studies [22,23,24,28,30], and as high in two studies [25,29]. With respect to missing outcome data, nine studies [22,23,24,25,26,27,28,30,31] were judged to be at low risk of bias, whereas two studies [29,32] were classified as raising some concerns. All included studies [22,23,24,25,26,27,28,29,30,31,32] were judged to be at low risk of bias in the domains of measurement of the outcome and selection of the reported result. The individual domain assessments and the overall risk-of-bias judgments for the included studies are summarized in Figure 2 and Figure 3.

### 3.4. Digital Technology Tools and Healthcare Professionals’ Role

The interventions were delivered remotely using a range of digital health technologies, including web-based programs [22,25,31], mobile phones [26,29], wearable devices [27], smartphone applications [22,23,28,30], and mini-apps [32]. These approaches were designed to deliver and evaluate the effectiveness, feasibility, safety, and clinical utility of remotely delivered interventions in adults with CVDs.

Across the studies, different levels of user support were provided. In five studies, participants received personalized intervention [22,29] and feedback [23,25,27,28,30]. In addition, five studies [23,26,27,30,32] included self-monitoring features, allowing participants to track their own health data and progress.

The involvement of health professionals also varied. In two RCTs [29,31], a multidisciplinary team of healthcare professionals participated in the delivery of the interventions. In other studies, the intervention was guided by a smaller number of specialists, such as cardiologists and physiologists [26], dietitians-nutritionists [12,22,23,33], and physiotherapists [25,32], depending on the program’s focus.

### 3.5. Effects on Primary Outcomes

Evidence suggests that remotely delivered nutrition with or without PA interventions significantly improved dietary behaviors, including increased consumption of fruits and vegetables and reduced salt intake, thereby enhancing overall diet quality [23,24,29,30,31,32] in patients with CVDs. Similarly, adherence to the MD was significantly greater in the IG compared with the CG in two studies [23,30].

Regarding cardiovascular outcomes, one study [23] reported a significantly lower heart rate in the IG compared with the CG. In contrast, findings related to BP were less consistent. Four studies [24,28,29,30] found no significant between-group differences, whereas only one study [23] demonstrated a significant reduction in BP following the intervention.

No significant effects were observed for most anthropometric measures, including BMI [22,23,25,29,30], body weight [28], waist circumference [23,30], and body fat percentage [24]. A significant reduction was observed in the IG in body water when comparing the final and first assessments [27]. Similarly, no significant between-group differences were reported for major biochemical markers, including total cholesterol [23,30], LDL-C [22,23,28,29,30], HDL-C [22,23,28,30], and HbA1c [22,23,30]. However, one study [23] reported significantly lower blood glucose levels in the IG compared with the CG.

The findings regarding triglyceride concentrations [23,28] and functional capacity, assessed by the 6-Minute Walk Distance (6MWD) [23,31], were inconsistent across studies. Furthermore, no significant differences between groups were observed in aerobic capacity, as measured by peak oxygen uptake (VO_2peak_) [22,28].

PA levels were significantly higher in the IG in six studies [23,25,28,29,30,32], whereas one study [31] reported no significant between-group differences. With respect to QoL, two studies [25,32] demonstrated significant improvements in overall QoL, while four studies [22,26,28,31] found no significant effects. Additionally, one study [30] reported a significant improvement only in the physical component of QoL.

Clinical outcomes were assessed less frequently. One study [27] evaluated rehospitalization rates, urgent physician visits, and mortality, reporting no significant differences between groups. Likewise, another study [30] found no significant differences in the incidence of acute cardiovascular events.

### 3.6. Effects on Secondary Outcomes

Regarding secondary outcomes, several studies [23,24,26,27,29] assessed the usability, acceptability, safety, and adherence of the remote interventions. Overall, the findings suggested favorable participant perceptions, high adherence rates, and good acceptability of the intervention protocols. Usability was evaluated in three studies [23,26,29]. Participants generally reported positive experiences with the interventions, describing them as useful [26] and, in one study, as excellent overall [23]. Only one study [24] reported no adverse events during the study period. Adherence to the interventions was assessed in two studies [26,27], both of which reported adherence rates of approximately 80%. Acceptability was evaluated in two studies [26,29]. In one study [26], participants reported that the intervention was well integrated into their daily lives (mean score: 11.7 ± 3.05) (Table 3). Likewise, the SMS-based intervention demonstrated high acceptability, with 82.0% of participants reporting that the messages were useful and 94.6% indicating that they were easy to understand [29].

## 4. Discussion

### 4.1. Overview and Interpretation of Main Findings

**Figure 4:**
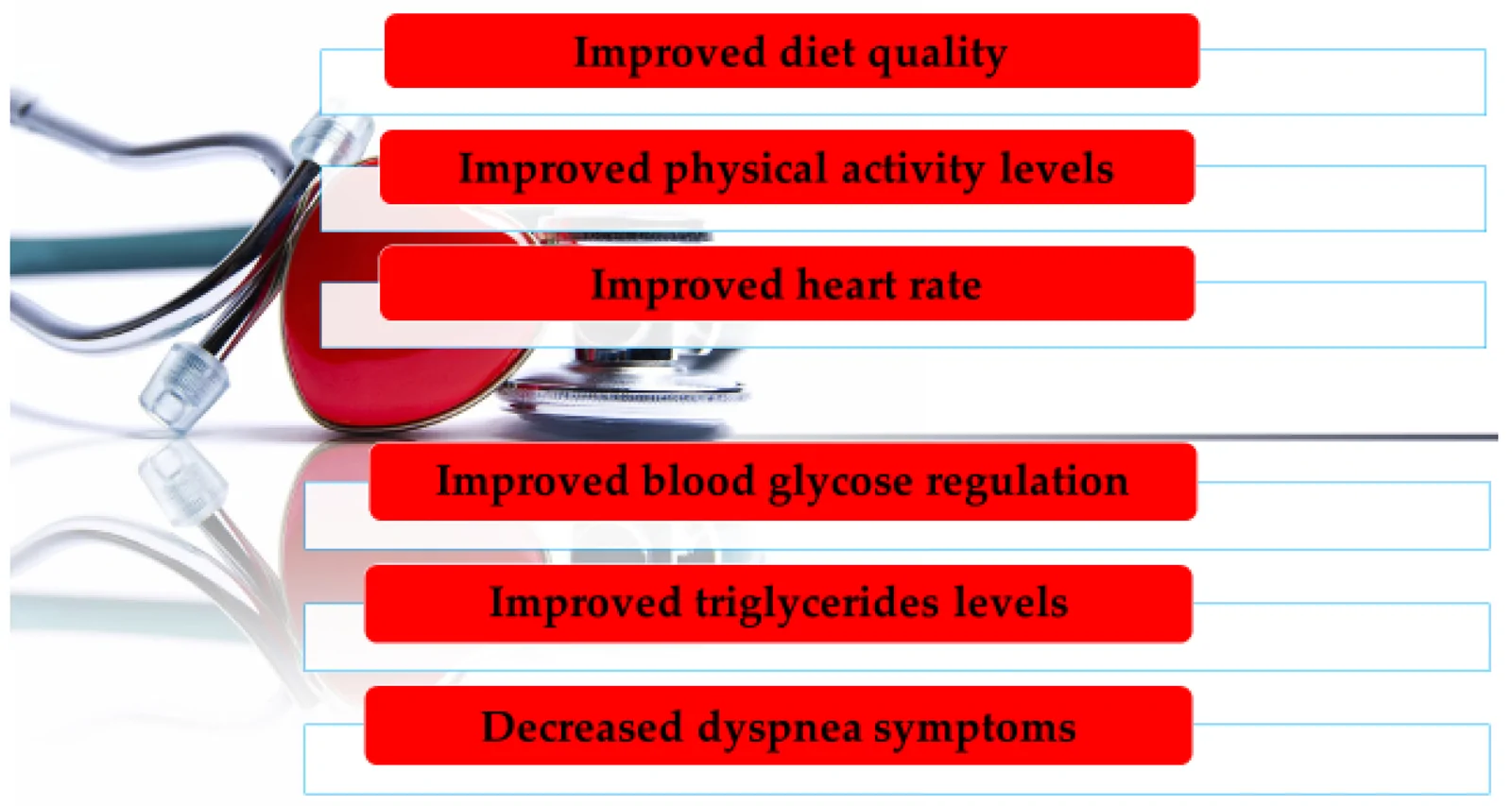
Effects of combined remotely delivered nutrition interventions with physical activity interventions on clinical outcomes.

This systematic review synthesized evidence from 11 RCTs [22,23,24,25,26,27,28,29,30,31,32] investigating remote nutrition interventions with or without PA interventions in adults with CVDs. Across the included studies, interventions were delivered through web-based platforms, text messages, mobile applications, and wearable monitoring devices. Collectively, the available evidence indicates that remotely delivered interventions were effective, feasible, and safe [12,13,14,22,23,24,25,26,27,28,29,30,31,32,33,34,35,36,37,38]. Overall, remote interventions improved diet quality [23,24,30,31,32], blood glucose regulation [23], heart rate [23], and PA levels [23,25,28,29,30,32] among patients with CVDs. Conversely, no significant difference was reported in aerobic capacity [22,28], functional capacity [23,31], BMI [22,23,29,30,32], or waist circumference [23,30]. As concerns another biochemical biomarker, decreased values of triglycerides were found, and, specifically, 0.4 mg/dL was the difference between the intervention and the control group in one trial [28]. Additionally, another important finding is decreased dyspnea symptoms [27] and decreased level of nicotine dependence [30] or smoking abstinence (33%) [30]. A summary of the favorable effects observed across the included studies is presented in Table 4 and Figure 4. The reported values originate from individual studies and are presented descriptively rather than as combined statistical effect estimates. Nevertheless, these findings support the potential role of remotely delivered interventions in the management of CVDs, the interpretation of these results should be considered in light of the substantial heterogeneity across the included studies.

The favorable effects may be attributable to enabling healthcare providers to monitor PA, dietary habits, and clinical indicators while offering individualized feedback and motivation [25,39,40]. Digital interventions may improve healthy behaviors (increased PA [12,41,42], improved diet quality [12], and reduced sedentary time [42]), and are even more effective when used to address multiple behavioral outcomes [37]. However, the effects of remote interventions appear not to improve all unhealthy behaviors, such as alcohol consumption [12], although they appeared effective for smoking cessation [43,44] and stress reduction [45].

The observed findings of combined nutrition and PA interventions in the included RCTs may be explained by the fact that digital health interventions primarily target behavioral determinants rather than directly influencing physiological outcomes. Interventions incorporating smartphone applications, web-based platforms, text messaging, and remote monitoring facilitate self-management through evidence-based behavior change techniques, including goal setting, self-monitoring, personalized feedback, reminders, and motivational support [25,26,27,28,29,30,31,32]. These features enhance patients’ self-efficacy, engagement, and adherence to recommended lifestyle behaviors, thereby explaining the consistent improvements in PA and dietary habits reported across several studies [25,29,30,32]. In contrast, the absence of significant effects on clinical parameters, such as BMI, blood pressure, lipid profiles, and cardiovascular events, is likely attributable to the multifactorial nature of these outcomes, which are influenced by genetic predisposition, disease severity, pharmacological treatment, and long-term lifestyle patterns. Moreover, many of the included interventions were of relatively short duration and may not have provided sufficient time for sustained behavioral changes to translate into measurable physiological improvements [25,26,27,32]. The widespread provision of guideline-directed usual care in the control groups may also have reduced the incremental benefits of the digital interventions, making between-group differences more difficult to detect. Furthermore, several studies included modest sample sizes and were not statistically powered to identify differences in relatively infrequent outcomes such as rehospitalization or mortality [27,30]. Collectively, these findings suggest that digital interventions are effective as behavioral support strategies but should be considered complementary to comprehensive cardiovascular care rather than standalone interventions capable of producing immediate clinical benefits.

Regarding the nutrition interventions, the findings of these studies suggest that digital dietary interventions are effective in improving adherence to recommended nutritional behaviors, but their effects on anthropometric and cardiometabolic outcomes remain limited [22,23,24]. This discrepancy may be explained by the ability of digital technologies to facilitate behavior change through personalized education, real-time feedback, self-monitoring, goal setting, and motivational strategies, all of which are established behavior change techniques that enhance adherence and self-management. Consequently, interventions delivered through smartphone applications and web-based platforms consistently improved adherence to the MD, reduced the consumption of unhealthy foods, increased healthy food intake, and promoted physical activity [22,23,24]. However, improvements in dietary behaviors do not necessarily translate into immediate changes in clinical outcomes such as BMI, blood pressure, lipid profile, or glycemic control. These physiological parameters are influenced by multiple interacting factors, including medication use, baseline cardiovascular risk, genetic predisposition, disease severity, and the cumulative effects of long-term lifestyle habits. Moreover, several interventions were of relatively short duration (4–24 weeks) [23,24,25,26,27,29,32], which may have been insufficient for sustained dietary modifications to produce measurable metabolic or anthropometric changes. The provision of comprehensive guideline-based care to participants in the control groups may have further attenuated between-group differences, particularly for well-controlled cardiovascular risk factors. Nevertheless, the significant reductions in systolic blood pressure, heart rate, blood glucose, and improvements in functional capacity observed in the eMOTIVA intervention [23] indicate that more comprehensive, interactive, and behaviorally tailored digital interventions may achieve broader clinical benefits when they successfully promote sustained lifestyle changes and high levels of patient engagement.

Current evidence suggests that education alone appeared less effective than combined educational and behavioral support programs [46]; however, this finding was not confirmed in another RCT [25]. Notably, MD compliance increased significantly from 18.4% to 57.1% for traditional coaching and from 27.5% to 64.7% for remote diet consulting [38], highlighting the pivotal role of dietitians. Beyond overall dietary quality, web-based and e-counseling interventions were associated with increased consumption of fruit and vegetable intake [25], improved CHD self-care behaviors [31], medication adherence, and PA levels compared with controls [29]. These findings are clinically important considering the poor baseline adherence to dietary recommendations among individuals with CVDs; in one study, 71% (59/83) of patients did not consume at least five portions of fruit and vegetables daily [25]. However, improvements in dietary behaviors were not consistently demonstrated across all studies, as one recent trial reported no improvement in dietary habits [32]. Participants in the IG were more likely to consume fruit and vegetables frequently, adhere to medication, and increase PA compared with controls [25,29]. In terms of nutrients, the IG significantly increased their intake of omega-3 and fiber, and decreased their intake of carbohydrates and saturated fatty acids compared with the CG [22]. Beckie et al. and Duan et al. reported improvements in eating habits, confidence, and self-efficacy for disease management [25,46]. On the contrary, Engelen et al. observed no changes in unhealthy dietary habits, possibly due to substantial missing data or the multifactorial nature of dietary behavior [36,47]. Nutrition knowledge and diet quality improved significantly in the TeleDiet group compared with the CG [33]. In a remote, web-based ancillary study using weighed 7-day diet records, adherence to the Portfolio Diet increased in both patients with high cardiovascular risk or CVD in the app group and in the CG. However, no significant between-group differences were observed [48]. Better diet quality was also associated with weight loss [13]. Overall, these findings support the integration of telehealth-based programs into standard care [26]. The heterogeneity in outcomes observed across the included studies is likely multifactorial and may explain why some remotely delivered dietary interventions achieved clinically meaningful improvements while others demonstrated limited effectiveness. Intervention intensity and duration appear to be important determinants of success, as programs that provided more frequent contact, individualized dietary counseling, goal setting, and ongoing feedback generally reported greater improvements in dietary behaviors and cardiovascular risk factors than lower-intensity interventions with minimal follow-up. Participant adherence also played a critical role, as sustained engagement with digital platforms and consistent participation in counseling sessions were associated with better outcomes, whereas declining engagement over time may have attenuated intervention effects. In addition, digital literacy and access to technology likely influenced participants’ ability to effectively use remote platforms, particularly among older adults who comprise a substantial proportion of individuals with CVDs. Variations in disease severity, comorbidity burden, baseline dietary habits, and motivation for behavior change may have further contributed to differences in responsiveness to the interventions. Finally, methodological differences across studies, including intervention components, outcome measures, follow-up duration, and study quality, likely contributed to the observed heterogeneity. These findings suggest that remotely delivered nutritional interventions are most effective when they are individualized, of sufficient intensity and duration, supported by strategies to promote long-term engagement, and tailored to the clinical and technological needs of people living with CVDs.

### 4.2. Comparison with Previous Literature

Several studies have compared adherence rates between home-based rehabilitation and center-based rehabilitation [17,40,49,50,51]. Diet quality improved across several studies through greater adherence to MD [22,23,30,33] and Portfolio dietary patterns [52], improved Nutri-scores [33], and healthier eating behaviors [43], including higher fruit and vegetable intake and lower salt consumption [24,53]. Beyond dietary improvements, several studies reported favorable effects on psychological parameters, including a statistically significant reduction in depressive symptoms reported by Lundgren et al. (2016) [54]. One study in women with CHD reported improvements in waist circumference, weight, and BMI [55]. These results may be attributable to adherence to healthy dietary habits, including higher intake of olive oil (4 spoons/day), fruits (≥3 servings/day), beans (1 cup/week), fish (3 times/week), vegetables (≥2 times/day), and whole-meal cereals (≥1 time/day), and lower intake of red meat (<30 g/day), sweets (<2 cookies/week), and sugar-sweetened beverages (<100 mL/day) [23,43]. In particular, polyphenol-rich diets, as described above, have been associated with health benefits, improved cardiovascular health, and reduced all-cause mortality [56,57]. Potential explanatory mechanisms include protection against free-radical damage through antioxidant-rich compounds, support for synaptic health and detoxification pathways relevant to non-communicable diseases [58], and interactions with the microbiota [59]. In addition, the European Food Safety Authority (EFSA) considers a daily fiber intake above 25 g sufficient to reduce cardiometabolic complications, including CHD, and support weight maintenance and postprandial glucose control [60]. Maintenance of energy balance may also contribute to these effects [61]. Dalleck et al. (2011) showed that energy expenditure increased significantly from baseline in both the conventional and telemedicine-delivered groups, reaching 1181 and 1225 kcal/week, respectively, by the end of the program [47]. Importantly, achieving an energy expenditure of at least 1000 kcal/week was associated with a 10–20% reduction in the risk of CHD events [62]. Visualized interventions (comics/videos/images) resulted in greater monthly improvements in healthy diet (β = 0.41, 95% CI [0.25–0.57]) and medication adherence (β = 0.38, 95% CI [0.22–0.54]), while the text message interventions showed greater improvement in regular exercise (β = 0.35, 95% CI [0.20–0.50]) [35].

Exercise-only interventions consistently improved functional capacity, symptom burden, and health-related QoL across cardiovascular populations [31]. Combined nutrition advice and exercise interventions, although less frequently studied, provided additional benefits, including improved body composition, cardiometabolic risk profiles, and long-term adherence [25,39,40]. In particular, these interventions improved lipid profiles, including the total cholesterol/HDL-C ratio, an important predictor of mortality risk [12,63]. These findings suggest that remote delivery models can successfully replicate the clinical outcomes of traditional center-based rehabilitation while enhancing accessibility and patient engagement [64]. However, in the My Heart Mate study, 24.4% of participants discontinued using the app due to difficulty with it. Some participants also reported increased anxiety related to app use and requested more personalized applications tailored to their health profile, risk factors, goals, and preferences [65].

Remote diet programs based on the MD [22,23,30,33] and Portfolio dietary patterns [48,66] have shown promising results in patients with CVD [22,23,24,33,38]. The MD has consistently been associated with reduced cardiovascular events and mortality, particularly in the PREDIMED trial [67]. The Portfolio diet has been shown to significantly lower LDL-C levels, with effects comparable to those of first-generation statins [48,66]. Studies included in this review suggest that digitally delivered dietary interventions can improve diet quality, lipid profiles, and cardiometabolic risk factors in CVD populations while enhancing accessibility and long-term engagement [12,13,14,22,23,24,25,26,27,28,29,30,31,32,33,34,35,36,38,40]. A recent study reported increased PA levels in 82% of patients with CAD and improved dietary habits in 59% [68]. Text-message interventions increased PA levels, whereas website interventions were more effective in promoting dietary change [29,35]. Together, these findings support the integration of Mediterranean [23,33,38] and Portfolio dietary patterns into remote nutrition programs as effective evidence-based components of secondary prevention strategies for CVD [68].

In patients with CVDs, the combination of a healthy diet and regular PA improves outcomes through several complementary mechanisms. More specifically, the MD or Dietary Approaches to Stop Hypertension (DASH) diet reduces intake of saturated fats, refined sugars, and excess sodium, while increasing fiber, antioxidants, and unsaturated fats [69]. These dietary modifications have been associated with lower LDL-C, improved HDL-C levels, reduced BP, and decreased systemic inflammation and oxidative stress, thereby contributing to the progression of atherosclerosis [70]. Conversely, regular PA (e.g., aerobic exercise) enhances endothelial function, improves insulin sensitivity, promotes weight control, lowers resting BP, and increases cardiac efficiency. Exercise also stimulates nitric oxide production, improving vascular dilation and blood flow [71]. These lifestyle interventions reduce the risk of thrombosis, stabilize atherosclerotic plaques, improve lipid and glucose metabolism, and ultimately decrease the likelihood of adverse cardiovascular events, such as myocardial infarction and stroke [72]. A healthy diet, according to Hull et al. (2025) [69], either as part of a personalized diet plan or just nutrition health advice, could have a beneficial effect on patients with CVD because it improves signaling pathways and so allows communication with the host’s heart and blood vessels [70].

Given the established benefits of lifestyle modification, telehealth-delivered CR incorporating structured exercise, nutrition education, risk-factor management, and behavioral support, has been extensively studied [13,47,73,74]. These studies showed effects on blood pressure, lipid profile, BMI, and functional capacity comparable to those of center-based programs, and in some studies, achieved better outcomes than usual care [13,75]. For example, the iCARE program led to greater improvements in healthy eating, medication adherence, and smoking reduction, whereas text-message interventions were more effective at increasing regular PA [35]. The greatest benefits were observed around six months, with medication adherence reaching approximately 12% above the control group, although substantial individual variation in behavioral change remained, especially for smoking [35]. Higher engagement was associated with greater improvements in cardiovascular risk factors over 18 months, whereas low engagement was associated with no significant improvements compared with controls [76]. Early engagement, personalization, regular two-way contact, and measurable exercise monitoring were associated with better outcomes, increased participation, and greater adherence while maintaining clinical effectiveness [74,77].

Our findings align with previous systematic reviews demonstrating that multimodal home-based CR programs with structured follow-up, monitoring, and behavioral support following cardiac events can achieve outcomes comparable to those of supervised programs [51]. In CVD populations, digital lifestyle interventions consistently improved exercise adherence, functional capacity, and metabolic risk factors, although one study found no effect on mortality [78]. Although fewer high-quality RCTs have combined remote nutrition and exercise interventions, emerging evidence supports this integrated approach [12,13,14,25,26,27,28,29,30,31,32,34,35,36,40]. A recent multicenter RCT in patients with CHD and type 2 diabetes showed that a telemedicine-supported lifestyle intervention improved glycemic control compared with usual care [79]. Similar improvements in fasting glucose and HbA1c were reported in a text-message intervention [80]. Pilot and feasibility studies suggest that multimodal remote lifestyle programs may improve self-management, exercise self-efficacy, and adherence, particularly when supported by behavioral engagement strategies [81]. These findings support the combination of diet and exercise in remote interventions to improve clinical and behavioral outcomes.

Evidence from both RCTs and cohort studies indicates that remote CR and lifestyle interventions in cardiovascular populations [26,82]. Exercise-only remote programs improved exercise capacity, physical activity, and adherence, with outcomes comparable to traditional facility-based rehabilitation [64]. Combined dietary and exercise interventions have been studied more extensively in CVD populations [12,13,14,35] than in other chronic diseases [83]. These interventions were associated with improved cardiometabolic risk factors, including lipid profiles, BP, and overall cardiovascular risk, as well as greater long-term adherence [12,13,34,38]. These interventions also improved weight loss and may reduce cardiovascular-related emergency department visits and rehospitalizations after percutaneous coronary intervention for acute coronary syndrome [12].

Across cardiovascular management, exercise-only interventions reliably improved functional outcomes and symptoms [84]. A recent hybrid comprehensive tele rehabilitation intervention in patients with HF with exercise training compared with usual care was effective at 9 weeks, significantly improving peak oxygen consumption (0.95 [95% CI, 0.65–1.26] mL/kg/min vs. 0.00 [95% CI, −0.31 to 0.30] mL/kg/min; *p* <  0.001) and QoL (Medical Outcome Survey Short Form–36 questionnaire score, 1.58 [95% CI, 0.74–2.42] vs. 0.00 [95% CI, −0.84 to 0.84]; *p* =  0.008). It was well tolerated, with no serious adverse events during exercise [85]. Although exercise-inclusive interventions may report adverse events [37], serious intervention-related adverse events were generally rare [86,87,88].

Beyond clinical effectiveness, combined nutritional and PA interventions reported benefits included reductions in mortality, improvements in cost-effectiveness (40%), and overall health outcomes (35%) [89]. Weng et al. (2025) reported that both app use and improvement in left ventricular ejection fraction (LVEF) were associated with lower overall mortality [90]. Smartphone-based applications may therefore support patients during transitional care [90]. Adherence rates were generally high in remote IGs, with several studies reporting participation rates equal to or greater than those in center-based programs [91,92].

### 4.3. Strengths and Limitations

This review has several strengths. First, it followed PRISMA guidelines and included only RCTs, thereby strengthening the methodological rigor of the findings. Second, it provided a comprehensive evaluation of both intervention efficacy and real-world feasibility. Subgroup analyses according to intervention modality (nutrition interventions alone versus combined nutrition and PA interventions) enabled a more nuanced interpretation of the available evidence. Furthermore, the focus on remotely delivered interventions is particularly relevant given the increasing implementation of telehealth and digital health technologies in cardiovascular rehabilitation [93].

Despite the strengths, several limitations should be acknowledged. First, the number of eligible studies [12,13,14,25,26,27,28,29,30,31,32,34,35,36,37] was limited, particularly those [22,23,24,33,38] evaluating nutrition interventions as standalone components, reflecting an important gap in the current literature. Existing evidence suggests that multimodal interventions may provide broader benefits, especially among patients with multimorbidity, underscoring the need for additional high-quality RCTs in this area [93]. Second, substantial heterogeneity was observed across studies in intervention characteristics, duration, follow-up periods, and outcome measures, precluding a quantitative meta-analysis. Variability in the reporting of adherence and the use of digital technologies further limited direct comparisons between studies. Additionally, the long-term sustainability of intervention effects remains uncertain, as intervention durations varied considerably, ranging from four weeks [24,27] to 12 months [28,31], although one study reported follow-up extending to five years [28]. Finally, although only randomized controlled trials were included in this review, the overall risk of bias was judged to be low in only two studies [26,31], indicating that methodological concerns were present in the majority of the included trials. Therefore, the interpretation of the findings should be approached with caution, as the presence of methodological uncertainties may affect the internal validity of the included studies and potentially influence the certainty and robustness of the overall conclusions. 

### 4.4. Future Directions and Clinical Implications

Further research should focus on the development and evaluation of personalized remote interventions, including behavioral support, digital engagement strategies, and integration into routine care. Future trials should prioritize standardized intervention protocols, longer follow-up periods, and clinically meaningful outcomes, while also evaluating cost-effectiveness, patient-reported outcomes, and implementation strategies. In addition, the incorporation of objective monitoring tools, artificial intelligence-based personalization, and hybrid models combining remote and in-person care may further optimize the scalability and effectiveness of digital cardiovascular rehabilitation programs.

The long-term sustainability of remotely delivered nutritional interventions is a key consideration for their integration into routine cardiovascular disease management. From a clinical perspective, remotely delivered exercise programs serve as effective alternatives to center-based rehabilitation, particularly for patients facing mobility, geographic, or socioeconomic barriers [15]. Their flexibility, accessibility, and potential to reduce geographical and logistical barriers may facilitate continued patient engagement, particularly for individuals with limited access to in-person services. Integrating dietary counseling into remote rehabilitation may further enhance treatment effectiveness by favorable changes in body composition and cardiometabolic outcomes [12]. Clinicians and dietitians should consider integrating remotely delivered nutritional counseling into comprehensive cardiovascular rehabilitation programs, using evidence-based dietary recommendations tailored to patients’ clinical characteristics, preferences, and digital literacy. Adherence may be supported by combining nutritional counseling with structured physical activity programs, regular follow-up, and behavioral coaching delivered through digital platforms. These multidisciplinary approaches may improve patient engagement and facilitate long-term lifestyle changes. However, sustaining intervention effects depends on several factors, including ongoing patient engagement, regular reinforcement of behavior change, and integration into existing healthcare systems. Evidence from digital health and chronic disease management suggests that interventions incorporating personalized feedback, goal setting, self-monitoring, automated reminders, and periodic contact with healthcare professionals are more likely to promote long-term adherence than interventions relying solely on passive educational content. Furthermore, reimbursement policies, clinician training, and equitable access to digital technologies are essential to ensure that remote nutritional interventions can be implemented and maintained at scale. Future research should prioritize longer follow-up periods and implementation studies to evaluate not only the durability of clinical benefits but also patient engagement, cost-effectiveness, and the feasibility of integrating these interventions into routine cardiovascular care.

## 5. Conclusions

The available evidence suggests that remotely nutrition interventions, implemented either as standalone strategies or as components of multimodal lifestyle programs incorporating PA, may represent a feasible and potentially beneficial approach for supporting dietary management and selected cardiovascular health outcomes in individuals with CVDs. Improvements have been reported in diet quality, PA levels, blood glucose regulation, heart rate, and patient engagement, while telehealth-based approaches may help overcome barriers to participation in conventional center-based rehabilitation programs.

However, these findings should be interpreted with caution because the current evidence is based on a relatively small number of studies, most of which were judged to have some concerns or high risk of bias. Furthermore, substantial heterogeneity in intervention characteristics, duration, and outcome measures limits the certainty and generalizability of the available evidence. Consequently, the current literature does not permit definitive conclusions regarding the effectiveness of remote nutrition interventions or to identify the most effective intervention components. Future well-designed, adequately powered randomized controlled trials using standardized intervention protocols, consistent outcome measures, and longer follow-up periods are needed to establish the long-term effectiveness, implementation, and cost-effectiveness of these interventions. Further research should also explore personalized, culturally adaptable, and scalable remote nutrition strategies to optimize patient engagement and clinical outcomes facilitating their integration into routine cardiovascular care.

## Figures and Tables

**Table 1:** Characteristics of the included RCTs evaluating combined remotely delivered nutrition and physical activity interventions.

Authors (Year) and Country	CVD	Sample Size (*n*), Age, years: Mean (SD) Male/ Female %	Technology/Digital Tools	Intervention Group Program (Duration, Weeks)	Control Group Program (Duration, Weeks)	Outcomes Measures	Main Findings
Duan et al. (2018), China [25]	CHD	*n* = 114 IG: 60→44 45.8 ± 14.68 45.6/54.4% CG: 54→39 51.57 ± 11.57 48.1%/51.9%	Web-based intervention	Web-based health program about PA & FVC (8 weeks)	Usual care (no treatment) (8 weeks)	PA, FVC, BMI, QoL	IG vs. CG: Significantly more PA Significantly improved QoL No significant time × treatment interaction for BMI
Broers et al. (2020), Spain & Netherlands [26]	CAD, symptomatic HF, Hypertension	*n* = 149 63.57 ± 8.30 66.4/33.6% (HF: *n* = 36, CAD: *n* = 40, Hypertension *n* = 73) (71.3/28.7%) IG: 63.26 ± 8.35 70.3/29.7% CG: 63.88 ± 8.30 62.7/37.3%	Text messages via mobile phones	Health behavior change program [Do something Different], the Moves app and the Care Portal (to register the GPS location) and the Care Portal (home monitoring device measuring daily symptoms and electrocardiogram). (12 weeks)	Usual care (12 weeks)	Lifestyle (PA, nutrition, etc.) and QoL	QoL: No significant difference between the groups at 6 months. 82.4% completed the intervention using the digital tool. The intervention was perceived to be useful (13.88 ± 3.96). The intervention was integrated relatively well in participants’ lives (11.71 ± 3.05).
Yoon et al. (2024), Republic of Korea [27]	Hospitalized for Acute HF	*n* = 77→74 IG: 37 58.4 ± 16.0 64/36% CG: 37 65.7 ± 12.6 61/39%	Smartphone app and Bluetooth-connected monitoring devices	Report vital signs, symptoms, diet, medications, exercise regimen, and connection to Bluetooth devices. Participants received feedback or alerts (4 weeks)	Report vital signs, symptoms, diet, medication, exercise regimen without connecting Bluetooth devices. Participants could not receive any feedback or alerts (4 weeks)	Dyspnea, app adherence, Death, rehospitalization, urgent HF visits	IG vs. CG: App adherence, based on log-in access, was significantly higher (*p* = 0.003), 80% for IG vs. 39% for CG. No significant differences for death, rehospitalization, or urgent HF visits (*p* > 0.99). Significantly greater change in dyspnea symptom score (*p* = 0.048). A significant reduction was found in body water composition after 4 weeks in the IG (*p* = 0.003).
Lunde et al. (2025), Norway [28]	Patients with CVD after CR For ACS	*n* = 113 59.0 ± 8.7 (77.9%/22.1%) IG: 57→50 59.5 ± 9.1 (84.2/15.8%) CG: 56→51 58.4 ± 8.2 (71.4/28.6%)	Smartphone app	Create and set goals, with tasks and automatic reminders, and received individualized feedback through the app (1 year)	Usual care (1 year)	Aerobic capacity (VO_2peak_), exercise performance, body weight, BP, lipid profile (LDL-C, HDL-C), exercise habits (exercise sessions/week over past year), health-related QoL, health status (EQ-5D), cardiac events, physical activity (IPAQ)	IG vs. CG: No significant difference for VO_2peak_, BP, LDL-C, HDL-C, body weight, QoL Statistically significant differences were also observed in triglycerides (*p* = 0.048) in favor of the CG and exercise habits (*p* = 0.04) in favor of the IG, with 0.67 exercise sessions per week (95% CI 0.04–1.29). In the follow-up, 9 cardiac events were recurrent or new CAD, 3 patients experienced other heart-related events, and 2 patients in the IG underwent angiography.
Bae et al. (2021), Republic of Korea [29]	CHD underwent PCI for the first time	*n* = 879 60.4 ± 10.5 83.3/16.7% IG: *n* = 440→377 60.1± 10.6 83.6/16.4% CG: *n* = 439→350 60.7 ± 10.4 82.9/17.1%	Website and SMS or app text messages	Information about healthy diet, PA, smoking cessation, and cardiovascular health via Website and 4 text messages/week and usual care (24 weeks)	Usual care (24 weeks)	LDL-C levels, SBP, BMI, self-reported PA, diet	IG vs. CG PA was significantly higher by 220 min/week (95% CI 36–404). Participants in the IG were more likely to eat fruit or vegetables frequently (≥2 times a day) compared to those in the CG. No significant difference for LDL-C levels, SBP, or BMI. 82% found messages useful.
Bernal-Jiménez et al. (2024), Spain [30]	CAD underwent PCI	*n* = 128 59.49 ± 8.97 71.9/28.1% IG: *n* = 67 57.70 ± 8.167 79/21% CG: *n* = 67→61 61.46 ± 9.476 64/36%	Smartphone app	App providing education, self-monitoring, motivation to improve and maintain lifestyle habits, and weekly feedback (36 weeks)	Usual care (36 weeks)	Adherence to the MD, Frequency of food consumption, PA, QoL (SF-12), Well-being, BMI, WC, SBP, DBP, Heart Rate, HbA1c, Lipids, adverse cardiovascular events	IG vs. CG: Adherence to the MD was significantly higher in the IG vs. CG (11.83 ± 1.74 vs. 10.14 ± 2.02, *p* = 0.001). Significant reduction in the frequency of consumption of red meat (95% vs. 77%, *p* = 0.001) and industrial pastries (93% vs. 91%, *p* = 0.003). Significantly higher intake of vegetables (75% vs. 49%, *p* = 0.03), fruits (90% vs. 68%, *p* = 0.02), and whole-meal cereals (64% vs. 35%, *p* = 0.003). Significantly greater time doing PA (619.14 ± 318.21 vs. 471.70 ± 261.43, *p* = 0.007). Significantly greater physical component of QoL (45.80 ± 10.79 vs. 41.40 ± 10.78, *p* = 0.02). No significant difference between IG and CG for BMI, WC, SBP, DBP, HR, HbA1c, total cholesterol, LDL-C, HDL-C, triglycerides, HbA1c, adverse cardiovascular events, or the mental component of QoL.
Nolan et al. (2021), Canada [31]	CHF with reduced ejection fraction	*n* = 242 IG: *n* = 120→100 60 (52–69) (79/21%) CG: *n* = 122→98 59 (52–70) (75/25%)	E-mail and hyperlinks to multi-site	Internet-based e-counseling included consulting in diet, exercise, medication, smoke-free living, weight monitoring through motivational and cognitive-behavioral tools and usual care (12 months)	e-based conventional CHF self-care education and usual care (12 months)	QoL (KCCQ), functional capacity (6MWD), PA (4-day step count), FVC (Diet History Questionnaire)	Non-significant difference between groups in QoL, functional capacity, PA. Significant difference between groups in FVC (*p* = 0.04).
Duan et al. (2025), China [32]	CVD	*n* = 124 41.60 ± 13.48 (38.7/61.3%) IG: *n* = 62 41.13 ± 13.11 (40.3/59.7%) CG: *n* = 62 42.06 ± 13.93 (37.1/62.9%)	Mini app	Health behavior change program Mini app through a WeChat mini program [eHealth Homeland] related to moderate to vigorous PA and FVC, data collection, and usual care (10 weeks)	Usual care (10 weeks)	Adherence to recommended PA and FVC, depressive symptoms (Center for Epidemiological Studies-Depression), QoL (World Health Organization QoL-BREF)	IG vs. the CG. Medium to large effect sizes for moderate to vigorous PA (Cohen dT1T2 = 0.57, Cohen dT1T3 = 0.60), FVC (Cohen dT1T2 = 0.88, Cohen dT1T3 = 0.85), and the integrated lifestyle indicator (Cohen dT1T2 = 0.68, Cohen dT1T3 = 0.76). Small to medium effect sizes for changes in perceived QoL (Cohen dT1T2 = 0.37, Cohen dT1T3 = 0.28).

App: Application; IG: intervention group; ΒΜΙ: body mass index; BP: blood pressure; CAD: coronary artery disease; CG: control group; CHD: coronary heart disease; CHD: coronary heart disease; CVD: cardiovascular disease; CVRFs: cardiovascular risk factors; DBP: diastolic blood pressure; EQ-5D: EuroQol 5-Dimension; FVC: Fruit and vegetable consumption; HbA1c: glycated hemoglobin; HF: heart failure; IPAQ: International Physical Activity Questionnaire; KCCQ: Kansas city cardiomyopathy questionnaire; LDL-C: Low-density-lipoprotein cholesterol; MD: Mediterranean Diet; *n*: number; PA: physical activity; PCI: percutaneous coronary intervention; QoL: quality of life; SBP: systolic blood pressure; 6MWD: six minute walking distance; VO_2peak_: peak oxygen consumption; vs.: versus; WC: waist circumference.

**Table 2:** Characteristics of the included RCTs evaluating remotely delivered nutritional advice and/or diet interventions.

Study (Author, Year, Country)	CVD	Sample Size (*n*) Age, Years: Mean (SD) Male/ Female %	Technology/Digital Tools	Intervention Group Program (Duration, Weeks)	Control Group Program (Duration, Weeks)	Outcomes Measures	Main Findings
Goni et al. (2020), Spain [22]	Atrial tachyarrhythmia recurrence after catheter ablation in patients with AF	*n* = 720 IG *n* = 365→191 59.9 ± 10.5 (77.5/22.5%) CG *n* = 355→178 59.6 ± 10.9 (74.9/25.1%)	Websites and smartphone app	Remote nutritional-MD enriched with extra virgin olive oil (12 weeks) (2-year follow-up)	Usual care (12 weeks) (2-year follow-up)	MD score (MedDietScore), Nutrition-Score, nutritional score, HbA1c, LDL, HDL, BMI, aerobic capacity (VO_2peak_) and QoL.	IG vs. CG Mediterranean diet score was greater, but not significant (2.0 [−1.0, 4.0] vs. 0.0 [−3.0, 1.5], *p* = 0.066). The difference in the Nutrition-Score was significantly greater (3.0 [1.0, 3.5] vs. 0.0 [−3.0, 2.0], *p* = 0.029). No significant difference between groups for QoL, VO_2peak_ (*p* = 0.83), BMI (*p* = 0.679), HbA1c (*p* = 0.457), LDL (*p* = 0.689), HDL (*p* = 0.303)
Cruz-Cobo et al. (2024), Spain [23]	CAD patients who underwent PCI with stent implantation after ACS	*n* = 300 62.53 ± 8.65 (69/31%) IG *n* = 150→143 61.13 ± 8.69 (68.7/31.3%) CG *n* = 150→141 63.93 ± 8.41 (69.3/30.7%)	Smartphone app [eMOTIVA]	The app incorporates a virtual classroom providing audio and video information about a healthy lifestyle, a section for self-recording cardiovascular risk factors, and a section for feedback messages and gamification (24 weeks)	Usual care (24 weeks)	Adherence to the MD, frequency of food consumption, level of PA (METs/week and min/week), sedentary time (hours sitting/week), functional capacity (6-MWD), smoking cessation and nicotine dependence, level of knowledge acquired about CVRFs, app satisfaction and usability, BMI and WC, SBP, DBP, HR, total cholesterol, LDL-C, triglyceride, HbA1c, blood sugar	IG vs. CG Adherence to the MD was significantly higher in the IG than in the CG after both 3 months (mean 11.63 ± 1.70 points vs. mean 9.32 ± 2.55 points; *p* < 0.001). The consumption of red meat and industrial pastries was significantly lower in the IG than in the CG at 3 months (*p* < 0.001) and 6 months (*p* < 0.001). The consumption of oily fish, vegetables, fruit, and whole-meal cereals was significantly higher in the IG than in the CG at 3 months (*p* < 0.001) and at 6 months (*p* < 0.001). SBP was significantly lower in the IG than in the CG at both 3 months (128.96 ± 15.87 mmHg vs. 133.27 ± 14.85 mmHg; *p* = 0.01) and 6 months (130.00 ± 21.90 mmHg vs. 135.78 ± 16.73 mmHg; *p* = 0.01). HR was significantly lower in the IG than in the CG at 3 months (66.75 ± 8.91 beats/min vs. 71.93 ± 9.86 beats/min; *p* < 0.001) but not at 6 months. Blood sugar levels were significantly lower in the IG than in the CG at 6 months (101.10 ± 18.57 mg/dL vs. 115.44 ± 39.46 mg/dL; *p* = 0.007). Participants in the IG did significantly more PA than the CG at 3 months (578.10 ± 326.14 min/week vs. 443.46 ± 278.11 min/week; *p* < 0.001) and at 6 months (614.51 ± 332.26 min/week vs. 408.40 ± 274.49 min/week; *p* < 0.001). Regarding the intensity of PA (METs/week), the IG performed more intense activity than the CG at 3 months (1991.74 ± 1176.71 METs/week vs. 1490.48 ± 925.89 METs/week; *p* < 0.001) and 6 months (mean 2112.66 ± 1196.67 METs/week vs. 1372.60 ± 944.62 METs/week; *p* < 0.001). The PA was of moderate intensity in both groups. 6MWD was significantly higher in the IG than in the CG (473.49 ± 102.28 m vs. 447.25 ± 93.68 m; *p* = 0.04). No significant differences between IG and CG for BMI, WC, DBP, TC, HDL-C, LDL-C, triglycerides, and HbA1c. Participants in the IG presented a high level of satisfaction with the app at 3 months (42.32 ± 5.96 points) and 6 months (42.53 ± 6.38 points). Usability was excellent (>80.3 points) at 3 months (95.75 ± 4.04 points) and 6 months (95.60 ± 4.03 points).
Eyles et al. (2020), New Zealand [24]	CVD	*n* = 66 patients 64 ± 7 (83/17%) IG *n* = 33 CG *n* = 33 & 12 household shoppers	Smartphone app	Smartphone app that enables lower salt food choices by scanning the barcode of a packaged food using the smartphone camera to receive an immediate interpretive [SaltSwitch] (4 weeks)	Usual care (4 weeks)	Adverse events, consumption of salt, total fat, saturated fat, protein, or total sugar, SBP, urinary sodium, usability and acceptability of the app	IG vs. CG Participants in the IG purchased significantly less salt (adjusted mean (95% CI); 0.7 (0.52 to 0.88) g/MJ) compared to the CG (adjusted mean (95% CI); 1.00 (0.80 to 1.20) g/MJ; group difference (95% CI) −0.30 (−0.58 to −0.03) g/MJ; *p* = 0.03. No significant differences between IG and CG for total fat, saturated fat, protein, total sugar, or SBP (−1.7 (−7.4 to 3.9) mmHg (*p* = 0.54). No serious adverse events. 87% of participants reported using the SaltSwitch app while shopping for household food during the intervention period. 75% of participants reported finding the SaltSwitch app very easy to use.

AF: atrial fibrillation; App: Application; ΒΜΙ: body mass index; BP: blood pressure; CG: control group; CAD: coronary artery disease; CHD: coronary heart disease; CI: confidence interval; CVD: cardiovascular disease; DBP: diastolic blood pressure; FVC: Fruit and vegetable consumption; g/MJ: grams per megajoules; HbA1c: glycated hemoglobin; HF: heart failure; IG: intervention group; LDL-C: Low-density-lipoprotein cholesterol; MD: Mediterranean Diet; METs: metabolic equivalents of task; mg: milligram; mmHg: millimeters of hydrargyrum; *n*: number; PA: physical activity; QoL: quality of life; SBP: systolic blood pressure; 6MWD: six minute walking distance; VO_2peak_: peak oxygen consumption; WC: waist circumference; vs.: versus.

**Table 3:** The effect of digital health interventions (smartphone applications, websites, text messaging, and web-based platforms) in promoting health behaviors.

Outcome	Overall findings
Physical activity (PA)	Improved in most studies using apps, websites, or text messaging [23,25,29,30,32]. Lunde et al., 2025 [28] showed improved exercise habits, whereas Nolan 2021 [31] found no significant difference.
Diet/Nutrition	Strong evidence for improved Mediterranean diet adherence, increased fruit and vegetable intake, reduced red meat and processed pastry consumption, and reduced salt intake.
Quality of life (QoL)	Mixed results. Significant improvements were reported in Duan et al., 2018 [25], Bernal-Jiménez et al., 2024 [30], and Duan et al., 2025 [32], whereas Broers et al., 2020 [26], Nolan et al., 2021 [31], Goni et al., 2020 [22], and Lunde et al., 2025 [28] reported no significant differences.
Anthropometric measures (BMI/WC)	Most studies found no significant changes in BMI or waist circumference.
Blood pressure	Generally unchanged, although Cruz-Cobo et al., 2024 [23] reported lower systolic blood pressure following the intervention.
Blood lipids	LDL-C and HDL-C were generally unchanged. Lunde et al., 2025 [28] found triglycerides favored the control group.
Clinical outcomes	No consistent reductions in mortality, rehospitalization, or cardiovascular events. Yoon et al., 2024 [27] reported no differences in death or HF rehospitalization.
App engagement/usability	High adherence and satisfaction were consistently reported (approximately 80–87% adherence/use in several studies).

**Table 4:** The changes in patients’ biochemical biomarkers, anthropometric measures, and habits originated from individual included studies.

Biochemical Biomarkers and Anthropometric Measures *** [Ref]	Mean Difference After the Nutrition Interventions Compared with the Control Ones in each Included Study	*p* Value *
Decreased values of triglyceride (mg/dL) [28]	0.4	0.048 *
Decreased glucose (mg/dL) [23]	1.1	0.88
Improved exercise habits (sessions per week) [28]	0.67	0.004 *
Increased values of physical activity (min/week) [30]	147.44	0.007 *
Increased values of physical activity (sitting/week) [23]	1.2	0.001 *
Increased exercise capacity (6-MWT) (meters) [23]	26.24	0.04 *
Improvement of QoL [25]	14	<0.001 *
Improved quality of diet with Mediterranean diet-adherence [22]	1.69	0.01 *
Improved quality of diet with Mediterranean diet adherence (%) [23]	34.1	0.001 *
Improved Coro Nutrition Prevention score [33]	3	0.02 *
Improved Mediterranean diet score [22]	2	0.06
Improved nutritional knowledge [23]	1.1	0.048 *
Reduced salt consumption per person per day [24]	0.7	0.001 *
Decreased dyspnea symptom scores from baseline [27]	1	0.048 *
Giving up smoking (%) [30]	33	0.01 *
Decreased the level of nicotine dependence [30]	1.34	0.02 *

*** QoL: Quality of life, which was assessed using the Minnesota Living with Heart Failure Questionnaire (MLHFQ), the World Health Organization’s Quality of Life questionnaire, and the Dartmouth format, 6-MWT: 6 min walk test, ref: reference * Statistically significant difference.

## Data Availability

No new data were created or analyzed in this study. Data sharing is not applicable to this article.

## Supplementary Materials

The following supporting information can be downloaded at https://www.mdpi.com/article/10.3390/healthcare14162523/s1, Table S1: Overview of electronic databases, search contributions, and rationale for selection. Table S2: Search strategy for each database (12 January 2026). Table S3: PRISMA 2020 Checklist. Table S4: PICOTS eligibility criteria of the study. Table S5: Inclusion and exclusion criteria of studies.

## References

[1] World Health Organization (2025). World Health Statistics 2025: Monitoring Health for the SDGs, Sustainable Development Goals.

[2] Tsao C.W., Aday A.W., Almarzooq Z.I., Anderson C.A., Arora P., Avery C.L., Baker-Smith C.M., Beaton A.Z., Boehme A.K., Buxton A.E. (2023). Heart disease and stroke statistics—2023 update: A report from the American Heart Association. Circulation.

[3] Mozaffarian D. (2016). Dietary and policy priorities for cardiovascular disease, diabetes, and obesity: A comprehensive review. Circulation.

[4] Cena H., Calder P.C. (2020). Defining a healthy diet: Evidence for the role of contemporary dietary patterns in health and disease. Nutrients.

[5] Caspersen C.J., Powell K.E., Christenson G.M. (1985). Physical activity, exercise, and physical fitness: Definitions and distinctions for health-related research. Public Health Rep..

[6] Anderson L., Thompson D.R., Oldridge N., Zwisler A.D., Rees K., Martin N., Taylor R.S. (2016). Exercise-based cardiac rehabilitation for coronary heart disease. Cochrane Database Syst. Rev. J. Am. Coll. Cardiol..

[7] AlHarbi S., Khan S.S., Moallem A.A., Allagani R.M., Al Sari R.R., Gubari M.S., Al Murad B.M., Kazi N., Fallatah W.S., Sideeque S. (2025). Cardiac Rehabilitation: A Literature Review of Benefits, Challenges, and Emerging Approaches. Cureus.

[8] Estruch R., Ros E., Salas-Salvadó J., Covas M.-I., Corella D., Arós F., Gómez-Gracia E., Ruiz-Gutiérrez V., Fiol M., Lapetra J. (2018). Primary prevention of cardiovascular disease with a Mediterranean diet supplemented with extra-virgin olive oil or nuts. N. Engl. J. Med..

[9] Chindhy S., Taub P.R., Lavie C.J., Shen J. (2020). Current challenges in cardiac rehabilitation: Strategies to overcome social factors and attendance barriers. Expert. Rev. Cardiovasc. Ther..

[10] Bomtempo A.P.D., Main E., de Melo Ghisi G.L. (2022). Remote exercise engagement among individuals with cardiovascular disease: A systematic review of barriers and facilitators. J. Cardiopulm. Rehabil. Prev..

[11] Kitsiou S., Thomas M., Marai G.E., Maglaveras N., Kondos G., Arena R., Gerber B. (2017). Development of an innovative mHealth platform for remote physical activity monitoring and health coaching of cardiac rehabilitation patients. 2017 IEEE EMBS International Conference on Biomedical & Health Informatics (BHI).

[12] Widmer R.J., Allison T.G., Lennon R., Lopez-Jimenez F., Lerman L.O., Lerman A. (2017). Digital health intervention during cardiac rehabilitation: A randomized controlled trial. Am. Heart J..

[13] Barnason S., Zimmerman L., Schulz P., Pullen C., Schuelke S. (2019). Weight management telehealth intervention for overweight and obese rural cardiac rehabilitation participants: A randomised trial. J. Clin. Nurs..

[14] Wang T.-C., Hsieh Y.-C., Yang C.-Y., Lee C.-M., Liu C.-Y., Chiou A.-F. (2026). Effects of a multimodal support intervention (the NICE-Support programme) on frailty and quality of life in patients with heart failure: A randomized controlled trial. Eur. J. Cardiovasc. Nurs..

[15] Omboni S., McManus R.J., Bosworth H.B., Chappell L.C., Green B.B., Kario K., Logan A.G., Magid D.J., Mckinstry B., Margolis K.L. (2020). Evidence and recommendations on the use of telemedicine for the management of arterial hypertension: An international expert position paper. Hypertension.

[16] McDonagh S.T., Dalal H., Moore S., Clark C.E., Dean S.G., Jolly K., Cowie A., Afzal J., Taylor R.S. (2023). Home-based versus centre-based cardiac rehabilitation. Cochrane Database Syst. Rev..

[17] Popiolek-Kalisz J. (2026). The role of nutrition in cardiovascular protection—Personalized versus universal dietary strategies. Trends Cardiovasc. Med..

[18] Sharma N., Kaushik P., Singh R., Gehlot A., Rathour N., Akram S.V. (2025). Integration of AI in healthcare systems—A discussion of the challenges and opportunities of integrating AI in healthcare systems for disease detection and diagnosis. AI in Disease Detection: Advancements and Applications.

[19] Page M.J., McKenzie J.E., Bossuyt P.M., Boutron I., Hoffmann T.C., Mulrow C.D., Shamseer L., Tetzlaff J.M., Akl E.A., Brennan S.E. (2021). The PRISMA 2020 statement: An updated guideline for reporting systematic reviews. BMJ.

[20] Bowen D.J., Kreuter M., Spring B., Cofta-Woerpel L., Linnan L., Weiner D., Bakken S., Kaplan C.P., Squiers L., Fabrigio C. (2009). How we design feasibility studies. Am. J. Prev. Med..

[21] Sterne J.A., Savovic J., Page M.J., Elbers R.G., Blencowe N.S., Boutron I., Cates C.J., Cheng H.-Y., Corbett M.S., Eldridge S.M. (2019). RoB 2: A revised tool for assessing risk of bias in randomised trials. BMJ.

[22] Goni L., de la O V., Barrio-López M.T., Ramos P., Tercedor L., Ibañez-Criado J.L., Castellanos E., Ibanez Criado A., Macias Ruiz R., García-Bolao I. (2020). A remote nutritional intervention to change the dietary habits of patients undergoing ablation of atrial fibrillation: Randomized controlled trial. J. Med. Internet Res..

[23] Cruz-Cobo C., Bernal-Jiménez M.Á., Calle G., Gheorghe L.L., Gutiérrez-Barrios A., Cañadas D., Tur J.A., Vázquez-García R., Santi-Cano M.J. (2024). Efficacy of a Mobile health app (eMOTIVA) regarding compliance with cardiac rehabilitation guidelines in patients with coronary artery disease: Randomized controlled clinical trial. JMIR mHealth uHealth.

[24] Eyles H., McLean R., Neal B., Jiang Y., Doughty R.N., McLean R., Ni Mhurchu C. (2020). A salt-reduction smartphone app supports lower-salt food purchases for people with cardiovascular disease: Findings from the SaltSwitch randomised controlled trial. Eur. J. Prev. Cardiol..

[25] Duan Y.P., Liang W., Guo L., Wienert J., Si G.Y., Lippke S. (2018). Evaluation of a web-based intervention for multiple health behavior changes in patients with coronary heart disease in home-based rehabilitation: Pilot randomized controlled trial. J. Med. Internet Res..

[26] Broers E.R., Kop W.J., Denollet J., Widdershoven J., Wetzels M., Ayoola I., Piera-Jimenez J., Habibovic M. (2020). A Personalized eHealth Intervention for Lifestyle Changes in Patients with Cardiovascular Disease: Randomized Controlled Trial. J. Med. Internet Res..

[27] Yoon M., Lee S., Choi J.Y., Jung M.-H., Youn J.-C., Shim C.Y., Choi J.-O., Kim E.J., Kim H., Yoo B.-S. (2024). Effectiveness of a Smartphone App–Based Intervention With Bluetooth-Connected Monitoring Devices and a Feedback System in Heart Failure (SMART-HF Trial): Randomized Controlled Trial. J. Med. Internet Res..

[28] Lunde P., Bye A., Grimsmo J., Pripp A.H., Ritschel V., Jarstad E., Nilsson B.B. (2025). Effects of Individualized Follow-Up with an App Postcardiac Rehabilitation: Five-Year Follow-Up of a Randomized Controlled Trial. J. Med. Internet Res..

[29] Bae J.-W., Woo S.-I., Lee J., Park S.-D., Kwon S.W., Choi S.H., Yoon G.-S., Kim M.-S., Hwang S.-S., Lee W.K. (2021). mHealth Interventions for Lifestyle and Risk Factor Modification in Coronary Heart Disease: Randomized Controlled Trial. JMIR mHealth uHealth.

[30] Bernal-Jiménez M.Á., Calle G., Gutiérrez Barrios A., Gheorghe L.L., Cruz-Cobo C., Trujillo-Garrido N., Rodríguez-Martín A., Tur J.A., Vázquez-García R., Santi-Cano M. (2024). Effectiveness of an Interactive mHealth App (EVITE) in Improving Lifestyle After a Coronary Event: Randomized Controlled Trial. JMIR mHealth uHealth.

[31] Nolan R.P., Ross H.J., Farkouh M.E., Huszti E., Chan S., Toma M., D’Antono B., White M., Thomas S., Barr S.I. (2021). Automated E-Counseling for Chronic Heart Failure. Circ. Heart Fail..

[32] Duan Y., Liang W., Guo L., Zhan H., Xia C., Ma H., Shang B., Wang Y., Yang M., Cheng S. (2025). Effectiveness of a WeChat Mini Program–Based Intervention on Promoting Multiple Health Behavior Changes Among Chinese Patients with Cardiovascular Diseases in Home-Based Rehabilitation: Randomized Controlled Trial. J. Med. Internet Res..

[33] Kaihara T., Falter M., Scherrenberg M., Xu L., Maes J., Meesen E., Dendale P. (2023). The impact of dietary education and counselling with a smartphone application on secondary prevention of coronary artery disease: A randomised controlled study (the TeleDiet study). Digit. Health.

[34] Fezza G.C., Sansone S., Nolan R.P. (2022). Therapeutic components of digital counseling for chronic heart failure. Front. Psychiatry..

[35] Hou Q., Wu Y. (2025). Abstract 4370709: Individualized mHealth Intervention and 12-Month Health Behavior Trajectories for Coronary Artery Disease: An Open-label, Multicenter Randomized Controlled Trial. Circulation.

[36] Engelen M.M., van Dulmen S., Puijk-Hekman S., Vermeulen H., Nijhuis-van der Sanden M.W.G., Bredie S.J.H., van Gaal B.G.I. (2020). Evaluation of a Web-Based Self-Management Program for Patients with Cardiovascular Disease: Explorative Randomized Controlled Trial. J. Med. Internet Res..

[37] Fernández-Pombo C.N., Aldama-López G., Lorenzo-Carpente M., López-Perez M., Marzoa-Rivas R., Rodríguez-Fernández J., Vázquez-Rodríguez J.M. (2026). A Remote Nursing-Guided Secondary Prevention Programme in Acute Coronary Syndrome. The SPRING Randomised Controlled Trial. J. Adv. Nurs..

[38] Choi B.G., Dhawan T., Metzger K., Marshall L., Akbar A., Jain T., Young H.A., Katz R.J. (2019). Image-based mobile system for dietary management in an American cardiology population: Pilot randomized controlled trial to assess the efficacy of dietary coaching delivered via a smartphone app versus traditional counseling. JMIR mHealth uHealth.

[39] Frederix I., Driessche N.V., Hansen D., Berger J., Bonne K., Alders T., Dendale P. (2015). Increasing the medium-term clinical benefits of hospital-based cardiac rehabilitation by physical activity telemonitoring in coronary artery disease patients. Eur. J. Prev. Cardiol..

[40] Avila A., Claes J., Goetschalckx K., Buys R., Azzawi M., Vanhees L., Cornelissen V. (2018). A randomized study of home-based training intervention with telemonitoring guidance in coronary artery disease patients-(TRiCH) Study. J. Med. Internet Res..

[41] Snoek J.A., Meindersma E.P., Prins L.F., Van’t Hof A.W., de Boer M.J., Hopman M.T., Eijsvogels T.M., de Kluiver E.P. (2021). The sustained effects of extending cardiac rehabilitation with a six-month telemonitoring and telecoaching programme on fitness, quality of life, cardiovascular risk factors and care utilisation in CAD patients: The TeleCaRe study. J. Telemed. Telecare.

[42] Maddison R., Pfaeffli L., Whittaker R., Stewart R., Kerr A., Jiang Y., Kira G., Leung W., Dalleck L., Carter K. (2015). A mobile phone intervention increases physical activity in people with cardiovascular disease: Results from the HEART randomized controlled trial. Eur. J. Prev. Cardiol..

[43] Akhu-Zaheya L.M., Shiyab W.e.Y. (2017). The effect of short message system (SMS) reminder on adherence to a healthy diet, medication, and cessation of smoking among adult patients with cardiovascular diseases. Intern. J. Med. Inform..

[44] Chow C.K., Redfern J., Hillis G.S., Thakkar J., Santo K., Hackett M.L., Jan S., Graves N., de Keizer L., Barry T. (2015). Effect of Lifestyle-Focused Text Messaging on Risk Factor Modification in Patients With Coronary Heart Disease: A Randomized Clinical Trial. JAMA.

[45] Jóźwik S., Cieślik B., Gajda R., Szczepańska-Gieracha J. (2021). Evaluation of the Impact of Virtual Reality-Enhanced Cardiac Rehabilitation on Depressive and Anxiety Symptoms in Patients with Coronary Artery Disease: A Randomised Controlled Trial. J. Clin. Med..

[46] Beckie T.M., Sengupta A., Dey A.K., Dutta K., Ji M., Chellappan S. (2024). A Mobile Health Behavior Change Intervention for Women with Coronary Heart Disease: A randomized controlled pilot study. J. Cardiopulm. Rehabil. Prev..

[47] Dalleck L.C., Schmidt L.K., Lueker R. (2011). Cardiac rehabilitation outcomes in a conventional versus telemedicine-based programme. J. Telemed. Telecare.

[48] Jenkins D.J., Jones P.J., Lamarche B., Kendall C.W., Faulkner D., Cermakova L., Gigleux I., Ramprasath V., de Souza R., Ireland C. (2011). Effect of a dietary portfolio of cholesterol-lowering foods given at 2 levels of intensity of dietary advice on serum lipids in hyperlipidemia: A randomized controlled trial. JAMA.

[49] Dalal H.M., Zawada A., Jolly K., Moxham T., Taylor R.S. (2010). Home based versus centre based cardiac rehabilitation: Cochrane systematic review and meta-analysis. BMJ.

[50] Li X., Xu S., Zhou L., Li R., Wang J. (2015). Home-based exercise in older adults recently discharged from the hospital for cardiovascular disease in China: Randomized clinical trial. Nurs. Res..

[51] Bosshard W., Seematter-Bagnoud L., Major K., Krief H., Büla C.J. (2024). Home-based Rehabilitation After Inpatient Rehabilitation: Utilization Rate and Characteristics of Referred Patients. Arch. Phys. Med. Rehabil..

[52] Kavanagh M.E., Chiavaroli L., Quibrantar S.M., Viscardi G., Ramboanga K., Amlin N., Paquette M., Sahye-Pudaruth S., Patel D., Grant S.M. (2025). Acceptability of a Web-Based Health App (PortfolioDiet.app) to Translate a Nutrition Therapy for Cardiovascular Disease in High-Risk Adults: Mixed Methods Randomized Ancillary Pilot Study. JMIR Cardio.

[53] Nundy S., Razi R.R., Dick J.J., Smith B., Mayo A., O’Connor A., Meltzer D.O. (2013). A Text Messaging Intervention to Improve Heart Failure Self-Management after Hospital Discharge in a Largely African-American Population: Before-After Study. J. Med. Internet Res..

[54] Lundgren J.G., Dahlström Ö., Andersson G., Jaarsma T., Kärner Köhler A., Johansson P. (2016). The Effect of Guided Web-Based Cognitive Behavioral Therapy on Patients with Depressive Symptoms and Heart Failure: A Pilot Randomized Controlled Trial. J. Med. Internet Res..

[55] Sengupta A., Beckie T., Dutta K., Dey A., Chellappan S. (2020). A mobile health intervention system for women with coronary heart disease: Usability study. JMIR Form. Res..

[56] Zhao C., Yang Y., Yang Y., Yang W., Mu L., Jia Y. (2025). Nonlinear relationship between the dietary inflammatory index (DII) and stroke risk in metabolically healthy obese individuals: An analysis of NHANES 1999-2023 data. Front. Nutr..

[57] Guasch-Ferré M., Willett W.C. (2021). The Mediterranean diet and health: A comprehensive overview. J. Ιntern Med..

[58] Schwingshackl L., Morze J., Hoffmann G. (2020). Mediterranean diet and health status: Active ingredients and pharmacological mechanisms. Br. J. Pharmacol..

[59] Fiebiger U., Bereswill S., Heimesaat M.M. (2016). Dissecting the interplay between intestinal microbiota and host immunity in health and disease: Lessons learned from germfree and gnotobiotic animal models. Eur. J. Microbiol. Immunol..

[60] Delcour J.A., Aman P., Courtin C.M., Hamaker B.R., Verbeke K. (2016). Prebiotics, Fermentable Dietary Fiber, and Health Claims. Adv. Nutr..

[61] Heymsfield S.B., Peterson C.M., Thomas D.M., Hirezi M., Zhang B., Smith S., Bray G., Redman L. (2017). Establishing energy requirements for body weight maintenance: Validation of an intake-balance method. BMC Res. Notes.

[62] Menotti A., Lanti M. (2008). An Italian chart for cardiovascular risk estimate including high-density lipoprotein-cholesterol. Dis. Manag. Health Outcomes.

[63] Frederix I., Caiani E.G., Dendale P., Anker S., Bax J., Böhm A., Cowie M., Crawford J., De Groot N., Dilaveris P. (2019). ESC e-Cardiology Working Group Position Paper: Overcoming challenges in digital health implementation in cardiovascular medicine. Eur. J. Prev. Cardiol..

[64] Thomas R.J., Beatty A.L., Beckie T.M., Brewer L.C., Brown T.M., Forman D.E., Franklin B.A., Keteyian S.J., Kitzman D.W., Regensteiner J.G. (2019). Home-Based Cardiac Rehabilitation: A Scientific Statement from the American Association of Cardiovascular and Pulmonary Rehabilitation, the American Heart Association, and the American College of Cardiology. JACC.

[65] Gallagher R., Neubeck L., Davis A., Redfern J., Parker H.M., Hyun K., Chow C., Celermajer D.S., Buckley T., Schumacher T. (2025). A Self-Administered Gamified Mobile Application for Secondary Prevention of Heart Disease in Patients Following a Cardiac Event (MyHeartMate): Process Evaluation from a Randomized Controlled Trial. Games Health J..

[66] Jenkins D.J., Kendall C.W., Marchie A., Faulkner D.A., Wong J.M., de Souza R., Emam A., Parker T.L., Vidgen E., Lapsley K.G. (2003). Effects of a dietary portfolio of cholesterol-lowering foods vs lovastatin on serum lipids and C-reactive protein. JAMA.

[67] Salas-Salvadó J., Becerra-Tomas N., García-Gavilán J.F., Bullo M., Barrubes L. (2018). Mediterranean diet and cardiovascular disease prevention: What do we know?. Prog. Cardiovasc. Dis..

[68] Eckardt I., Buschhaus C., Nickenig G., Jansen F. (2021). Smartphone-guided secondary prevention for patients with coronary artery disease. J. Rehabil. Assist. Technol. Eng..

[69] Hull S.C., Mszar R., Ostfeld R.J., Ferrucci L.M., Mucci L.A., Giovannucci E., Loeb S. (2025). Diet and Prevention of Cardiovascular Disease and Cancer: JACC: CardioOncology State-of-the-Art Review. J. Am. Coll. Cardiol. CardioOnc..

[70] Lu L., Jing W., Qian W., Fan L., Cheng J. (2024). Association between dietary patterns and cardiovascular diseases: A review. Curr. Probl. Cardiol..

[71] Bozkurt B., Fonarow G.C., Goldberg L.R., Guglin M., Josephson R.A., Forman D.E., Lin G., Lindenfeld J., O’Connor C., Panjrath G. (2021). Cardiac Rehabilitation for Patients with Heart Failure: JACC Expert Panel. JACC.

[72] Narkhede M., Pardeshi A., Bhagat R., Dharme G. (2024). Review on emerging therapeutic strategies for managing cardiovascular disease. Curr. Cardiol. Rev..

[73] Robinson S.A., Moy M.L. (2022). Promoting Exercise Training Remotely. Life.

[74] Isakadze N., Kim C.H., Marvel F.A., Ding J., MacFarlane Z., Gao Y., Spaulding E.M., Stewart K.J., Nimbalkar M., Bush A. (2024). Rationale and Design of the mTECH-Rehab Randomized Controlled Trial: Impact of a Mobile Technology Enabled Corrie Cardiac Rehabilitation Program on Functional Status and Cardiovascular Health. JAHA.

[75] Rawstorn J., Gant N., Direito A., Beckmann C., Maddison R. (2016). Telehealth exercise-based cardiac rehabilitation: A systematic review and meta-analysis. Heart.

[76] Coley N., Andre L., Hoevenaar-Blom M.P., Ngandu T., Beishuizen C., Barbera M., van Wanrooij L., Kivipelto M., Soininen H., van Gool W. (2022). Factors Predicting Engagement of Older Adults with a Coach-Supported eHealth Intervention Promoting Lifestyle Change and Associations Between Engagement and Changes in Cardiovascular and Dementia Risk: Secondary Analysis of an 18-Month Multinational Randomized Controlled Trial. J. Med. Internet Res..

[77] Gallegos-Rejas V.M., Rawstorn J.C., Gallagher R., Mahoney R., Thomas E.E. (2024). Key features in telehealth-delivered cardiac rehabilitation required to optimize cardiovascular health in coronary heart disease: A systematic review and realist synthesis. Eur. Heart J. Digit. Health.

[78] Lima H.J., Souza R., Santos A., Borges D.L., Guimaraes A.R.F., Ferreira G., Barros R.M., Cordeiro A.L.L. (2020). Virtual reality on pulmonary function and functional independence after coronary artery bypass grafting: Clinical trial. Am. J. Cardiovasc. Dis..

[79] Mueller S., Dinges S.M.T., Gass F., Fegers-Wustrow I., Treitschke J., von Korn P., Boscheri A., Krotz J., Freigang F., Dubois C. (2025). Telemedicine-supported lifestyle intervention for glycemic control in patients with CHD and T2DM: Multicenter, randomized controlled trial. Nat. Med..

[80] Huo X., Krumholz H.M., Bai X., Spatz E.S., Ding Q., Horak P., Zhao W., Gong Q., Zhang H., Yan X. (2019). Effects of Mobile Text Messaging on Glycemic Control in Patients with Coronary Heart Disease and Diabetes Mellitus: A randomized clinical trial. Circ. Cardiovasc. Qual. Outcomes.

[81] Huang X., Song X., Sun M., Hou Y., Nan J., Gao J., Fu S., Jiang Y. (2025). Effectiveness of digital co-creation platform in remote pulmonary rehabilitation for older adults with chronic obstructive pulmonary disease: A randomized controlled trial. Front. Public Health.

[82] Rao A., Zecchin R., Byth K., Denniss A.R., Hickman L.D., DiGiacomo M., Phillips J.L., Newton P.J. (2021). The role of lifestyle and cardiovascular risk factors in dropout from an Australian cardiac rehabilitation program. A longitudinal cohort study. Heart Lung Circ..

[83] Passantino A., Dalla Vecchia L.A., Corrà U., Scalvini S., Pistono M., Bussotti M., Gambarin F.I., Scrutinio D., La Rovere M.T. (2021). The Future of Exercise-Based Cardiac Rehabilitation for Patients with Heart Failure. Front. Cardiovasc. Med..

[84] McMahon J., Thompson D.R., Pascoe M.C., Brazil K., Ski C.F. (2021). *e*Health interventions for reducing cardiovascular disease risk in men: A systematic review and meta-analysis. Prev. Med..

[85] Piotrowicz E., Pencina M.J., Opolski G., Zaręba W., Banach M., Kowalik I., Orzechowski P., Szalewska D., Pluta S., Główczyńska R. (2020). Effects of a 9-Week Hybrid Comprehensive Telerehabilitation Program on Long-term Outcomes in Patients with Heart Failure: The Telerehabilitation in Heart Failure Patients (TELEREH-HF) Randomized Clinical Trial. JAMA Cardiol..

[86] Felix H.C., West D.S. (2013). Effectiveness of weight loss interventions for obese older adults. Am. J. Health Promot..

[87] Tandon P., Purdy G., Ismond K.P., Cruz C., Etruw E., Suderman K., Hyde A., Stickland M., Spence J.C., Lien D.C. (2022). Heal-me PiONEer (personalized online nutrition and exercise): An RCT assessing 2 levels of app-based programming in individuals with chronic disease. Contemp. Clin. Trials.

[88] Piera-Jiménez J., Winters M., Broers E., Valero-Bover D., Habibovic M., Widdershoven J., Folkvord F., Lupiáñez-Villanueva F. (2020). Changing the Health Behavior of Patients with Cardiovascular Disease Through an Electronic Health Intervention in Three Different Countries: Cost-Effectiveness Study in the Do Cardiac Health: Advanced New Generation Ecosystem (Do CHANGE) 2 Randomized Controlled Trial. J. Med. Internet Res..

[89] Kruse C.S., Krowski N., Rodriguez B., Tran L., Vela J., Brooks M. (2017). Telehealth and patient satisfaction: A systematic review and narrative analysis. BMJ Open.

[90] Weng S.-C., Lin W.-W., Huang J.-L., Chao C.-Y., Hsu C.-Y., Lin S.-Y. (2025). A smartphone model for post-acute care decreases all-cause mortality with improved left ventricular ejection fraction in patients hospitalized with heart failure in Taiwan. Maturitas.

[91] Cruz-Cobo C., Bernal-Jiménez M.Á., Vázquez-García R., Santi-Cano M.J. (2022). Effectiveness of mHealth Interventions in the Control of Lifestyle and Cardiovascular Risk Factors in Patients After a Coronary Event: Systematic Review and Meta-analysis. JMIR mHealth uHealth.

[92] Vegesna A., Tran M., Angelaccio M., Arcona S. (2016). Remote Patient Monitoring via Non-Invasive Digital Technologies: A Systematic Review. Telemed. e-Health.

[93] Duff O.M., Walsh D.M.J., Furlong B.A., O’Connor N.E., Moran K.A., Woods C.B. (2017). Behavior Change Techniques in Physical Activity eHealth Interventions for People With Cardiovascular Disease: Systematic Review. J. Med. Internet Res..

