# Supplementary material for: Remotely Delivered Nutrition Interventions with or Without Physical Activity Interventions in Adults with Cardiovascular Diseases: A Systematic Review of Randomized Controlled Trials
Source: Healthcare (Basel). 2026 Aug 13;14(16):2523. doi: 10.3390/healthcare14162523 (PMC13511904; doi:10.3390/healthcare14162523)
Supplement: Supplementary file 1 [file healthcare-14-02523-s001.zip › healthcare-4422425-supplementary.pdf]

Supplementary Table S1. Overview of Electronic Databases, Search Contributions, and Rationale for Selection

Electronic Database	Contribution to Study	Reason for Inclusion
PubMed	Provided a broad understanding of the interplay between diet, food consumption, dietary interventions, and health benefits; MeSH terms ensured precision in the search for relevant literature	Widely recognized as a premier biomedical database, frequently used for reviews in healthcare research
Web of Science	Enhanced the overall coverage of literature related to the nutrients in diet, dietary interventions, and their impact on health	Provides a multidisciplinary approach, covering a wide range of scientific disciplines
Scopus	Strengthened the evidence base by focusing on bioactive compounds in diets related to evidence-based interventions; MeSH terms ensured specificity in selecting relevant literature	Renowned for reviews and emphasizing evidence-based interventions in healthcare research

Supplementary Table S2. Search strategy for each database (12 January 2026)

**PubMed Search Formula via NLM**
**Results: 430**
("Heart diseases"[MeSH Terms] OR "ischemic heart disease"[Text Word] OR "heart failure"[Text Word] OR "coronary artery disease"[Text Word] OR "chronic heart failure"[Text Word] OR "arrhythmias"[Text Word] OR "heart transplantation"[Text Word] OR "cardiovascular diseases"[All Fields] OR "hypertension"[Text Word] OR "blood pressure"[Text Word]) AND ("rehabilitation mesh"[All Fields] OR "program"[Text Word] OR "intervention"[Text Word] OR "Rehabilitation"[Text Word] OR "exercise"[Text Word] OR "physical therapy"[Text Word] OR "physical activity"[Text Word] OR "non-pharmacological treatment"[Text Word] OR "6-minute walk test"[Text Word] OR "6MWT"[Text Word] OR "six-minute walk test"[Text Word] OR "3-minute walk test"[Text Word] OR "3MWT"[Text Word] OR "three-minute walk test"[Text Word] OR "2-minute walk test"[Text Word] OR "2MWT"[Text Word] OR "two-minute walk test"[Text Word] OR "incremental shuttle walk test"[Text Word] OR "weight"[Text Word] OR "body weight"[Text Word] OR "BMI"[Text Word] OR "body mass index"[Text Word] OR "body mass"[Text Word] OR "fat mass"[Text Word] OR "fat-free mass"[Text Word] OR "fat-free mass"[Text Word] OR "FFMI"[Text Word] OR "fat-free mass index"[Text Word] OR "lean mass"[Text Word] OR "lean body mass"[Text Word] OR "lipids"[Text Word] OR "lipid*"[Text Word] OR "cholesterol"[Text Word] OR "total cholesterol"[Text Word] OR "LDL"[Text Word] OR "LDL cholesterol"[Text Word] OR "HDL"[Text Word] OR "HDL cholesterol"[Text Word] OR "triglycerides"[Text Word] OR "dyslipidemia"[Text Word] OR "hyperlipidemia"[Text Word] OR "lipid profile"[Text Word]) AND ((("Diet"[MeSH Terms] AND "OR"[All Fields]) AND ("Diet"[MeSH Terms] OR "Diet"[All Fields])) AND "tw or"[All Fields]) AND ("Diet"[MeSH Terms] OR "Diet"[All Fields] OR "diets"[All Fields] OR "diet s"[All Fields] OR "dieted"[All Fields] OR "dieting"[All Fields])) AND "tw or"[All Fields]) AND ("Diet"[MeSH Terms] OR "Diet"[All Fields] OR "dietary"[All Fields] OR "dietaries"[All Fields])) AND "tw or"[All Fields]) AND ("nutrition s"[All Fields] OR "nutritional status"[MeSH Terms] OR ("nutritional"[All Fields] AND "status"[All Fields]) OR "nutritional status"[All Fields] OR "nutrition"[All Fields] OR "nutritional sciences"[MeSH Terms] OR ("nutritional"[All Fields] AND "sciences"[All Fields]) OR "nutritional sciences"[All Fields] OR "nutritional"[All Fields] OR "nutritionals"[All Fields] OR "nutritions"[All Fields] OR "nutritive"[All Fields])) AND "tw or"[All Fields]) AND ("nutrition s"[All Fields] OR "nutritional status"[MeSH Terms] OR ("nutritional"[All Fields] AND "status"[All Fields]) OR "nutritional status"[All Fields] OR "nutrition"[All Fields] OR "nutritional sciences"[MeSH Terms] OR ("nutritional"[All Fields] AND "sciences"[All Fields]) OR "nutritional sciences"[All Fields] OR "nutritional"[All Fields] OR "nutritionals"[All Fields] OR "nutritions"[All Fields] OR "nutritive"[All Fields])) AND "tw or"[All Fields]) AND ("eating"[MeSH Terms] OR "eating"[All Fields] OR ("dietary"[All Fields] AND "intake"[All Fields]) OR "dietary intake"[All Fields])) AND "tw or"[All Fields]) AND ("eating"[MeSH Terms] OR "eating"[All Fields] OR ("food"[All Fields] AND "intake"[All Fields]) OR "food intake"[All Fields])) AND "tw or"[All Fields]) AND ("eating"[MeSH Terms] OR "eating"[All Fields] OR ("nutrient"[All Fields] AND "intake"[All Fields]) OR "nutrient intake"[All Fields])) AND "tw or"[All Fields]) AND ("eating"[MeSH Terms] OR "eating"[All Fields])) AND "tw or"[All Fields])

AND ("feeding behavior"[MeSH Terms] OR ("feeding"[All Fields] AND "behavior"[All Fields]) OR "feeding behavior"[All Fields] OR ("eating"[All Fields] AND "habits"[All Fields]) OR "eating habits"[All Fields])) AND "tw or"[All Fields]) AND ("eating behaviour"[All Fields] OR "feeding behavior"[MeSH Terms] OR ("feeding"[All Fields] AND "behavior"[All Fields]) OR "feeding behavior"[All Fields] OR ("eating"[All Fields] AND "behavior"[All Fields]) OR "eating behavior"[All Fields])) AND "tw or"[All Fields]) AND ("feeding behavior"[MeSH Terms] OR ("feeding"[All Fields] AND "behavior"[All Fields]) OR "feeding behavior"[All Fields] OR ("dietary"[All Fields] AND "habits"[All Fields]) OR "dietary habits"[All Fields])) AND "tw or"[All Fields]) AND (("Diet"[MeSH Terms] OR "Diet"[All Fields] OR "dietary"[All Fields] OR "dietaries"[All Fields]) AND "pattern*"[All Fields])) AND "tw or"[All Fields]) AND (("food"[MeSH Terms] OR "food"[All Fields]) AND ("consumptions"[All Fields] OR "economics"[MeSH Terms] OR "economics"[All Fields] OR "consumption"[All Fields]))) AND "tw or"[All Fields]) AND (("food"[MeSH Terms] OR "food"[All Fields]) AND "choice*"[All Fields])) AND "tw or"[All Fields]) AND ("eating"[MeSH Terms] OR "eating"[All Fields] OR ("nutritional"[All Fields] AND "intake"[All Fields]) OR "nutritional intake"[All Fields])) AND "tw or"[All Fields]) AND ("nutritional status"[MeSH Terms] OR ("nutritional"[All Fields] AND "status"[All Fields]) OR "nutritional status"[All Fields])) AND "tw or"[All Fields]) AND (("Diet"[MeSH Terms] OR "Diet"[All Fields]) AND ("qualities"[All Fields] OR "quality"[All Fields] OR "quality s"[All Fields]))) AND "tw or"[All Fields]) AND (("Diet"[MeSH Terms] OR "Diet"[All Fields] OR "dietary"[All Fields] OR "dietaries"[All Fields]) AND ("qualities"[All Fields] OR "quality"[All Fields] OR "quality s"[All Fields]))) AND "tw or"[All Fields]) AND "food pattern*"[Text Word]) OR "nutrition behavior"[Text Word] OR "mediterranean diet"[Text Word] OR "health behavior"[Text Word] OR "health behaviour"[Text Word]) AND ("Telemedicine"[MeSH Terms] OR "Digital Technology"[MeSH Terms] OR "Wearable Electronic Devices"[MeSH Terms] OR "Telemedicine"[Text Word] OR "mhealth"[Text Word] OR "m-health"[Text Word] OR "mobile health"[Text Word] OR "mobile application"[Text Word] OR "smartphone"[Text Word] OR "smart phone"[Text Word] OR "smartphone application"[Text Word] OR "cellphone"[Text Word] OR "cellular phone"[Text Word] OR "telerehabilitation"[Text Word] OR "tele-rehabilitation"[Text Word] OR "virtual rehabilitation"[Text Word] OR "remote rehabilitation"[Text Word] OR "remote intervention"[Text Word] OR "telehealth"[Text Word] OR "tele-health"[Text Word] OR "telemonitor"[Text Word] OR "ehealth"[Text Word] OR "e-health"[Text Word] OR "digital health"[Text Word] OR "mobile technolog*"[Text Word] OR "website"[Text Word] OR "web-site"[Text Word] OR "web-based"[Text Word] OR "tele-supervised"[Text Word] OR "teletherapy"[Text Word] OR "wireless technology"[Text Word] OR "telemetry"[Text Word] OR "videoconferencing"[Text Word] OR "home-based rehabilitation"[Text Word] OR "home exercise training"[Text Word] OR "tele-guidance"[Text Word] OR "e-coach"[Text Word] OR "e-coaching"[Text Word] OR "tele-advice"[Text Word] OR "games"[Text Word] OR "tele-physiotherapy"[Text Word] OR "tele-intervention"[Text Word] OR "teleintervention"[Text Word] OR "tele-education"[Text Word] OR "teletreatment"[Text Word] OR "teletraining"[Text Word] OR "telecoaching"[Text Word] OR "video games"[Text Word] OR "serious games"[Text Word] OR "wearable*"[Text Word] OR "wearable devices"[Text Word] OR "activity tracker"[Text Word] OR "sensor-based"[Text Word])
**Web of Science (WoS) Formula**

**Results: 1.136**
(TS= ("Heart diseases" OR "ischemic heart disease" OR "heart failure" OR "coronary artery disease" OR "chronic heart failure" OR "arrhythmias" OR "heart transplantation" OR "cardiovascular diseases" OR "hypertension" OR "blood pressure") AND TS= ("Rehabilitation" OR "program" OR "intervention" OR "exercise" OR "physical activity" OR "non-pharmacological treatment" OR "6-minute walk test" OR "6MWT" OR "six-minute walk test" OR "3-minute walk test" OR "3MWT" OR "three-minute walk test" OR "2-minute walk test" OR "2MWT" OR "two-minute walk test" OR "weight" OR "body weight" OR "BMI" OR "body mass index" OR "body mass" OR "fat mass" OR "fat-free mass" OR "fat free mass" OR "FFMI" OR "fat-free mass index" OR "lean mass" OR "lean body mass" OR "lipids" OR "lipid" OR "cholesterol" OR "total cholesterol" OR "LDL" OR "LDL cholesterol" OR "HDL" OR "HDL cholesterol" OR "triglycerides" OR "dyslipidemia" OR "hyperlipidemia" OR "lipid profile") AND TS= ("Diet" OR "diet" OR "diets" OR "dietary" OR "nutrition" OR "nutritional" OR "dietary intake" OR "food intake" OR "nutrient intake" OR "eating" OR "eating habits" OR "eating behavior" OR "dietary habits" OR "dietary pattern" OR "food consumption" OR "food choice" OR "nutritional intake" OR "nutritional status" OR "diet quality" OR "dietary quality" OR "food pattern" OR "nutrition behavior" OR "mediterranean diet" OR "health behavior" OR "health behaviour") AND TS= ("Telemedicine" OR "Digital Technology" OR "Wearable Electronic Devices" OR "Telemedicine" OR "mhealth" OR "m-health" OR "mobile health" OR "mobile application" OR "smartphone" OR "smart phone" OR "smartphone application" OR "cellphone" OR "cellular phone" OR "telerehabilitation" OR "tele-rehabilitation" OR "virtual rehabilitation" OR "remote rehabilitation" OR "remote intervention" OR "telehealth" OR "tele-health" OR "telemonitor" OR "ehealth" OR "e-health" OR "digital health" OR "mobile technolog" OR "website" OR "web-site" OR "web-based" OR "tele-supervised" OR "teletherapy" OR "wireless technology" OR "telemetry" OR "videoconferencing" OR "home-based rehabilitation" OR "home exercise training" OR "tele-guidance" OR "e-coach" OR "e-coaching" OR "tele-advice" OR "games" OR "tele-physiotherapy" OR "tele-intervention" OR "teleintervention" OR "tele-education" OR "teletreatment" OR "teletraining" OR "telecoaching" OR "video games" OR "wearable" OR "wearable devices" OR "activity tracker" OR "sensor-based"))
**Scopus Search Formula via ELSEVIER**
**Results: 646**
(TITLE-ABS-KEY("Heart diseases" OR "ischemic heart disease" OR "heart failure" OR "coronary artery disease" OR "chronic heart failure" OR "arrhythmias" OR "heart transplantation" OR "cardiovascular diseases" OR "hypertension" OR "blood pressure") AND TITLE-ABS-KEY("Rehabilitation" OR "program" OR "intervention" OR "exercise" OR "physiotherapy" OR "physical therapy" OR "physical activity" OR "non-pharmacological treatment" OR "6-minute walk test" OR "6MWT" OR "six-minute walk test" OR "3-minute walk test" OR "3MWT" OR "three-minute walk test" OR "2-minute walk test" OR "2MWT" OR "two-minute walk test" OR "weight" OR "body weight" OR "BMI" OR "body mass index" OR "body mass" OR "fat mass" OR "fat-free mass" OR "fat free mass" OR "FFMI" OR "fat-free mass index" OR "lean mass" OR "lean body mass" OR "lipids" OR "lipid" OR "cholesterol" OR "total cholesterol" OR "LDL" OR "LDL cholesterol" OR "HDL" OR "HDL cholesterol" OR "triglycerides" OR "dyslipidemia" OR "hyperlipidemia" OR "lipid profile") AND TITLE-ABS-KEY("Diet" OR "diet" OR "diets" OR "dietary" OR "nutrition" OR

"nutritional" OR "dietary intake" OR "food intake" OR "nutrient intake" OR "eating" OR "eating habits" OR "eating behavior" OR "dietary habits" OR "dietary pattern" OR "food consumption" OR "food choice" OR "nutritional intake" OR "nutritional status" OR "diet quality" OR "dietary quality" OR "food pattern" OR "nutrition behavior" OR "mediterranean diet" OR "health behavior" OR "health behaviour") AND TITLE-ABS-KEY("Telemedicine" OR "Digital Technology" OR "Wearable Electronic Devices" OR "Telemedicine" OR "mhealth" OR "m-health" OR "mobile health" OR "mobile application" OR "smartphone" OR "smart phone" OR "smartphone application" OR "cellphone" OR "cellular phone" OR "telerehabilitation" OR "tele-rehabilitation" OR "virtual rehabilitation" OR "remote rehabilitation" OR "remote intervention" OR "telehealth" OR "tele-health" OR "telemonitor" OR "ehealth" OR "e-health" OR "digital health" OR "mobile technolog" OR "website" OR "web-site" OR "web-based" OR "tele-supervised" OR "teletherapy" OR "wireless technology" OR "telemetry" OR "videoconferencing" OR "home-based rehabilitation" OR "home exercise training" OR "tele-guidance" OR "e-coach" OR "e-coaching" OR "tele-advice" OR "games" OR "tele-physiotherapy" OR "tele-intervention" OR "teleintervention" OR "tele-education" OR "teletreatment" OR "teletraining" OR "telecoaching" OR "video games" OR "wearable" OR "wearable devices" OR "activity tracker" OR "sensor-based"))

Supplementary Table S3. PRISMA 2020 checklist.

Section and Topic	Item #	Checklist item	Location where item is reported
**TITLE**			
Title	1	Identify the report as a systematic review.	Page 1
**ABSTRACT**			
Abstract	2	See the PRISMA 2020 for Abstracts checklist.	Page 1
**INTRODUCTION**			
Rationale	3	Describe the rationale for the review in the context of existing knowledge.	Pages 2-3
Objectives	4	Provide an explicit statement of the objective(s) or question(s) the review addresses.	Page 3
**METHODS**			
Eligibility criteria	5	Specify the inclusion and exclusion criteria for the review and how studies were grouped for the syntheses.	Page 4
Information sources	6	Specify all databases, registers, websites, organisations, reference lists and other sources searched or consulted to identify studies. Specify the date when each source was last searched or consulted.	Pages 4-5
Search strategy	7	Present the full search strategies for all databases, registers and websites, including any filters and limits used.	Table S2
Selection process	8	Specify the methods used to decide whether a study met the inclusion criteria of the review, including how many reviewers screened each record and each report retrieved, whether they worked independently, and if applicable, details of automation tools used in the process.	Pages 4-5
Data collection process	9	Specify the methods used to collect data from reports, including how many reviewers collected data from each report, whether they worked independently, any processes for obtaining or confirming data from study investigators, and if applicable, details of automation tools used in the process.	Pages 4-5
Data items	10a	List and define all outcomes for which data were sought. Specify whether all results that were compatible with each outcome domain in each study were sought (e.g. for all measures, time points, analyses), and if not, the methods used to decide which results to collect.	Pages 4-5
	10b	List and define all other variables for which data were sought (e.g. participant and intervention characteristics, funding sources). Describe any assumptions made about any missing or unclear information.	Pages 3-5
Study risk of bias assessment	11	Specify the methods used to assess risk of bias in the included studies, including details of the tool(s) used, how many reviewers assessed each study and whether they worked independently, and if applicable, details of automation tools used in the process.	Pages 4-5
Effect measures	12	Specify for each outcome the effect measure(s) (e.g. risk ratio, mean difference) used in the synthesis or presentation of results.	Pages 4-5
Synthesis methods	13a	Describe the processes used to decide which studies were eligible for each synthesis (e.g. tabulating the study intervention characteristics and comparing against the planned groups for each synthesis (item #5)).	Pages 3-4
	13b	Describe any methods required to prepare the data for presentation or synthesis, such as handling of missing summary statistics, or data conversions.	Page 4
	13c	Describe any methods used to tabulate or visually display results of individual studies and syntheses.	Pages 3-5
	13d	Describe any methods used to synthesize results and provide a rationale for the choice(s). If meta-analysis was performed, describe the model(s), method(s) to identify the presence and extent of statistical heterogeneity, and software package(s) used.	Pages 3-5
	13e	Describe any methods used to explore possible causes of heterogeneity among study results (e.g. subgroup analysis, meta-regression).	Pages 3-5
	13f	Describe any sensitivity analyses conducted to assess robustness of the synthesized results.	Pages 3-5
Reporting bias assessment	14	Describe any methods used to assess risk of bias due to missing results in a synthesis (arising from reporting biases).	Pages 3-5

Section and Topic	Item #	Checklist item	Location where item is reported
Certainty assessment	15	Describe any methods used to assess certainty (or confidence) in the body of evidence for an outcome.	Pages 3-5
**RESULTS**			
Study selection	16a	Describe the results of the search and selection process, from the number of records identified in the search to the number of studies included in the review, ideally using a flow diagram.	Page 5
	16b	Cite studies that might appear to meet the inclusion criteria, but which were excluded, and explain why they were excluded.	Page 5
Study characteristics	17	Cite each included study and present its characteristics.	Tables 1-2
Risk of bias in studies	18	Present assessments of risk of bias for each included study.	Page 13
Results of individual studies	19	For all outcomes, present, for each study: (a) summary statistics for each group (where appropriate) and (b) an effect estimate and its precision (e.g. confidence/credible interval), ideally using structured tables or plots.	Page 15 Table 3
Results of syntheses	20a	For each synthesis, briefly summarise the characteristics and risk of bias among contributing studies.	Page 13 Figures 2-3
	20b	Present results of all statistical syntheses conducted. If meta-analysis was done, present for each the summary estimate and its precision (e.g. confidence/credible interval) and measures of statistical heterogeneity. If comparing groups, describe the direction of the effect.	Page 17
	20c	Present results of all investigations of possible causes of heterogeneity among study results.	Page 17
	20d	Present results of all sensitivity analyses conducted to assess the robustness of the synthesized results.	Pages 16-18
Reporting biases	21	Present assessments of risk of bias due to missing results (arising from reporting biases) for each synthesis assessed.	Page 17
Certainty of evidence	22	Present assessments of certainty (or confidence) in the body of evidence for each outcome assessed.	
**DISCUSSION**			
Discussion	23a	Provide a general interpretation of the results in the context of other evidence.	Page 15
	23b	Discuss any limitations of the evidence included in the review.	Pages 21-22
	23c	Discuss any limitations of the review processes used.	Pages 21-22
	23d	Discuss implications of the results for practice, policy, and future research.	Page 22
**OTHER INFORMATION**			
Registration and protocol	24a	Provide registration information for the review, including register name and registration number, or state that the review was not registered.	
	24b	Indicate where the review protocol can be accessed, or state that a protocol was not prepared.	Page 3
	24c	Describe and explain any amendments to information provided at registration or in the protocol.	Pages 3-4
Support	25	Describe sources of financial or non-financial support for the review, and the role of the funders or sponsors in the review.	No financial support. Page 23
Competing interests	26	Declare any competing interests of review authors.	Page 23
Availability of data, code and	27	Report which of the following are publicly available and where they can be found: template data collection forms; data extracted from included studies; data used for all analyses;	Figure 1 Tables

Section and Topic	Item #	Checklist item	Location where item is reported
other materials		analytic code; any other materials used in the review.	S1-S2 and Tables S3-4

From: Page MJ, McKenzie JE, Bossuyt PM, Boutron I, Hoffmann TC, Mulrow CD, et al. The PRISMA 2020 statement: an updated guideline for reporting systematic reviews. BMJ 2021;372:n71. doi: 10.1136/bmj.n71. This work is licensed under CC BY 4.0. To view a copy of this license, visit https://creativecommons.org/licenses/by/4.0/

Supplementary Table S4. PICOTS eligibility criteria of the study

**P** (Population)	Adult patients (≥18 years) with Cardiovascular Diseases (coronary arterial diseases, acute and chronic heart failure, coronary artery bypass surgery, coronary heart disease, percutaneous coronary intervention; ACS: acute coronary syndrome, and/or mixed populations) were defined as the population.
**I** (Intervention)	Participants in the intervention group received remote nutrition counseling/advice/coaching, diet, with or without a combination of PA advice and/or constructed interventions.
**C** (Comparator)	Participants in the comparator group received in-person nutrition counseling/advice/coaching, diet, with or without a combination of PA advice and/or constructed interventions, usual care (e.g., pharmacological therapy), education, or no intervention.
**O** (Outcomes)	Primary outcomes: Biochemical biomarkers, total cholesterol, low-density lipoprotein-cholesterol, high-density lipoprotein-cholesterol, triglycerides, and non-high-density, lipoprotein-cholesterol, glucose, glycated hemoglobin , cardiovascular indexes (heart rate, systolic and diastolic blood pressure), anthropometric parameters (body mass index , waist circumference, waist-to-hip ratio, body composition), levels of physical activity, exercise and functional capacity, adherence to the Mediterranean Diet, diet quality, quality of life, rehospitalization, urgent visits, death and acute events.<br>Secondary outcomes: feasibility (implementation, demand, acceptability, practicality, safety, and clinical utility)
**T** (TimeFactor)	Any intervention time period
**S** (Study design)	Randomized controlled trial (RCT)

Supplementary Table S5. Inclusion and exclusion criteria of studies.

**Inclusion Criteria**	**Exclusion Criteria**
Published in English	Non- Randomized Control Trials (such as reviews, case reports, and practical guidelines)
Randomized controlled trials	Animal studies

Adult patients with CVDs	Studies without a Comparator Group (i.e., control or alternative dietary intervention)
Studies with a minimum of 4 weeks of follow-up and a minimum of 40 completers	Publication not available in English language
	Studies published before 2011
